# Comprehensive repertoire of the chromosomal alteration and mutational signatures across 16 cancer types

**DOI:** 10.1038/s41588-025-02474-x

**Published:** 2026-02-13

**Authors:** Andrew Everall, Avraam Tapinos, Aliah Hawari, Alex J. Cornish, Amit Sud, Daniel Chubb, Ben Kinnersley, Anna Frangou, Miguel Barquin, Josephine Jung, David N. Church, Ludmil B. Alexandrov, Richard S. Houlston, Andreas J. Gruber, David C. Wedge

**Affiliations:** 1 Division of Genetics and Epidemiology, The Institute of Cancer Research, London, UK; 2 Manchester Cancer Research Centre, University of Manchester, Manchester, UK; 3 UCL Cancer Institute, London, UK; 4 Nuffield Department of Medicine, Big Data Institute, University of Oxford, Oxford, UK; 5 Oxford NIHR Comprehensive Biomedical Research Centre, Oxford University Hospitals NHS Foundation Trust, Oxford, UK; 6 Department of Biology, University of Konstanz, Konstanz, Germany; 7 Department of Neurosurgery, King’s College Hospital NHS Foundation Trust, London, UK; 8 Institute of Psychiatry, Psychology & Neuroscience, King’s College London, London, UK; 9 Wellcome Trust Centre for Human Genetics, University of Oxford, Oxford, UK; 10 Department of Cellular and Molecular Medicine, University of California, San Diego, La Jolla, CA USA; 11 Department of Bioengineering, University of California, San Diego, La Jolla, CA USA; 12 Moores Cancer Center, University of California, San Diego, La Jolla, CA USA; 13 NIHR Manchester Biomedical Research Centre, Manchester, UK

**Keywords:** Cancer, Personalized medicine, Cancer, Genome informatics, Clinical genetics

## Abstract

Whole-genome sequencing (WGS) enables exploration of the full spectrum of oncogenic processes that generate characteristic patterns of mutations. Mutational signatures provide clues to tumor etiology and highlight potentially targetable pathway defects. Here alongside single-base substitution, doublet-base substitution, small insertion and deletion and copy number aberration signatures previously covered by the Catalogue of Somatic Mutations in Cancer (COSMIC), we report signatures from an additional mutation type, structural variations (SVs), extracted de novo from WGS in 10,983 patients across 16 tumor types recruited to the 100,000 Genomes Project. Across the five mutation classes, we report 134 signatures, 26 of which are new to COSMIC, including an SV signature reference set. By relating signatures to genomic features and clinical phenotypes, we provide further insights into mutagenic processes and the application of signature analysis to precision oncology.

## Abstract (web summary)

This study analyses whole-genome sequencing data of cancer samples from 10,983 patients and explores relationships among mutational signatures, various biomarkers and clinical outcomes.

## Main

**Extended Data Fig. 1:**
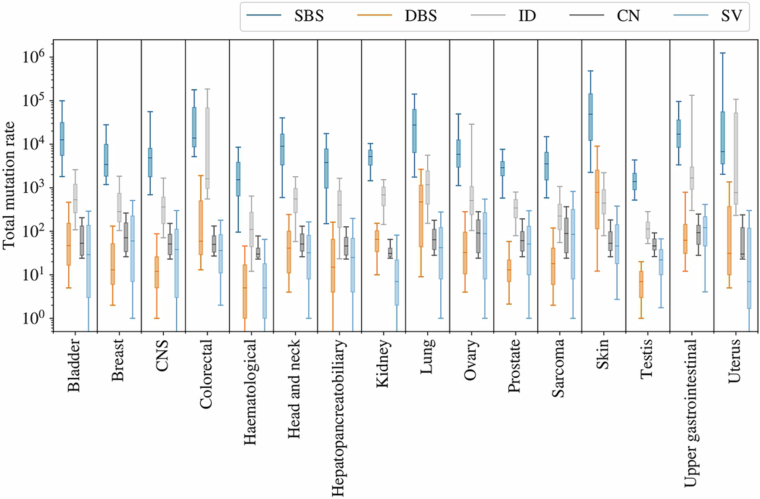
Distribution of total mutation rates. Total mutation rates are shown for each of the 5 mutation types analyzed in this work (SBS, DBS, ID, CN and SV) and each of the 16 tumor types. Each box and whisker show the median, 16–84 percentile range and 2.5–97.5 percentile range for samples in the tumor group.

Somatic mutations in cancer are a consequence of endogenous and exogenous processes^1–3^, each of which leaves a unique mutational signature through DNA damage, repair and replication. The genomic alterations observed in a cancer genome typically reflect multiple overlapping mutational signatures, which can be computationally extracted by breaking down mutation patterns across tumors into distinct components^4–6^. By 2023, the Catalogue of Somatic Mutations in Cancer (COSMIC; v3.3) cataloged 60 single-base substitution (SBS) signatures, many linked to specific mutational processes, although their growing number complicates fitting them to new data. Technical factors can also create artifactual signatures, adding further challenges and complicating assignment^7^. Whole-genome sequencing (WGS) may enable better separation of partially correlated signatures than exome sequencing^5,6^. Additionally, WGS enables signature types based on copy number (CN) and structural variation (SV) to be characterized^8,9^. Using WGS data from the 100,000 Genomes Project (100KGP), we analyzed 371,254,410 mutations from 10,983 patients across 16 cancer types (Extended Data Fig. 1), detailing mutational and chromosomal signatures. This work deepens insights into cancer-causing processes, connects signatures to clinical features and underscores their value in precision oncology.

## Results

### Signature analysis

**Fig. 1:**
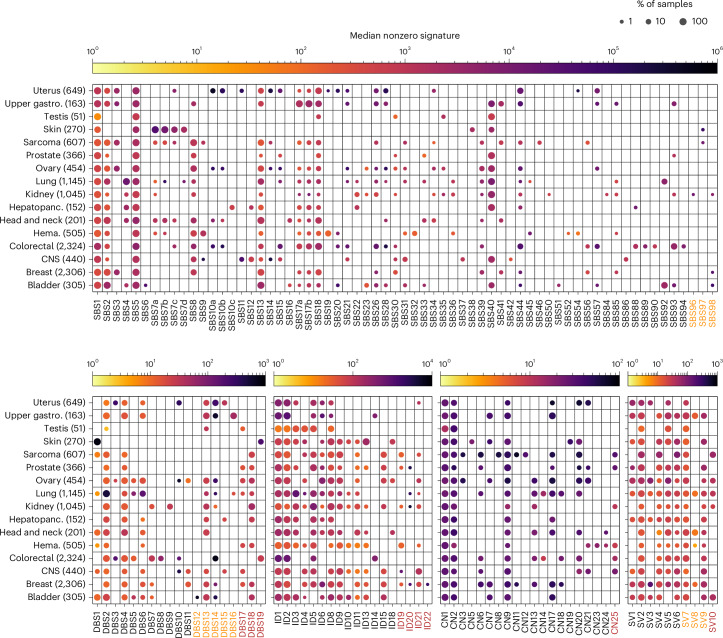
Signature activity across cancer types. Circle size corresponds to the percentage of samples in which the signature is nonzero, while its color corresponds to the median activity (that is, mutation/alteration burden, of nonzero activity samples). Orange signature labels indicate signatures not previously reported in COSMIC or in ref. ^11^ but are highly similar to signatures from previous publications^9,13^, while the red labeled signatures denote those that are new. For each signature type, only samples with $> 20$ mutations/alterations are included in the figure to reduce noise, as low mutation counts can lead to high uncertainty in signature activity assignment. Gastro., gastrointestinal; hepatopanc., hepatopancreatobiliary; hema., hematological.

**Fig. 2:**
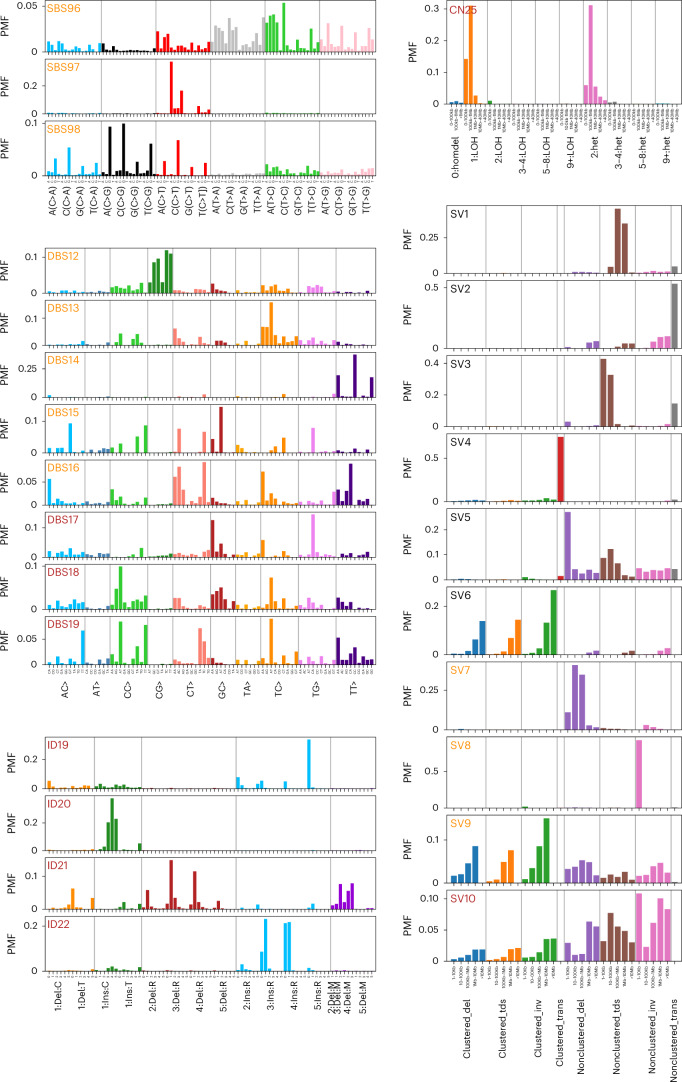
New SBS, DBS, ID, CN and ten SV signatures extracted de novo*.* The PMF is the fractional contribution of the given mutation type to the signature. SV1–SV6 are matched to the six signatures reported in ref. ^11^. PMF, probability mass fraction.

Using SigProfilerExtractor (SPE)^10^, SBS, doublet-base substitution (DBS) and small insertion and deletion (ID), CN aberration and SV signatures were extracted independently per tissue type. SBS signatures were expanded to 288 classes by considering the transcriptional context of mutations (that is, whether mutations fell on the transcribed, untranscribed or nontranscribed strand)^10^, while DBS, ID and CN mutational types were classified as per COSMIC. SV signatures, new to COSMIC, were categorized by type, size and clustering^9^. Across all cancers, 67 SBS, 19 DBS, 18 ID and 20 CN signatures were identified (Fig. 1), including 3 SBS, 8 DBS, 4 ID and 1 CN signatures not previously cataloged as well as 10 SV signatures new to COSMIC (Fig. 2). Of the signatures extracted, two COSMIC reference signatures (SBS24 and SBS29) have no activity in any samples (Supplementary Note 1).

### Characteristics of new signatures

Based on cosine similarity (cos(sim) > 0.8), SV signatures 1–6 have previously been reported in breast cancer^11^. While SV signatures 7–9 have been recovered in several different cancers^9^, SV10 is new (Fig. 2). SV1 and SV3 feature nonclustered tandem duplications (>100 kb and <100 kb, respectively). SV2 and SV4 consist of nonclustered and clustered translocations, while deletions define SV5 and SV7 (<10 kb and 10 kb to 1 Mb, respectively). SV8 is characterized by small inversions (<10 kb). SV6, SV9 and SV10 feature multiple different rearrangements, excluding translocations, with SV6 primarily composed of large (≥10 Mb) clustered deletions, tandem duplications and inversions, whereas SV9 is defined by clustered SVs of <10 Mb. SV10 is primarily composed of a range of nonclustered SVs, including smaller numbers of clustered SVs of ≥1 Mb.

SV2 and SV7 were ubiquitous (Fig. 1), and SV4 and SV6 occurred in all tissue types except uterine and testicular cancers. Except in sarcoma, SV9 tended to co-occur with SV4 and SV6, and SV5 was not identified in sarcoma or bladder cancer. SV1 and SV3 were prominent in breast, ovarian and uterine cancers; SV8 in head and neck, lung and upper gastrointestinal cancers and SV10 in sarcoma, bladder, kidney, lung and ovarian cancers.

SBS96, with a broad mutation profile, was only detected in a minority of kidney cancers. SBS97, characterized by C > T mutations and showing similarity to SBS7b, was a feature of skin cancer and sarcoma. SBS98, extracted in bladder, breast and kidney cancers, is dominated by mutations with NCG context (where N represents any of the bases A, C, G or T), similar to SBS87 (Fig. 2), which in acute lymphoblastic leukemia (ALL) is linked to thiopurine treatment^12^. SBS98 is mainly composed of C > G mutations in contrast to SBS87, which is characterized by C > T mutations. While new to COSMIC, SBS96, SBS97 and SBS98 have also been reported in ref. ^13^.

**Fig. 3:**
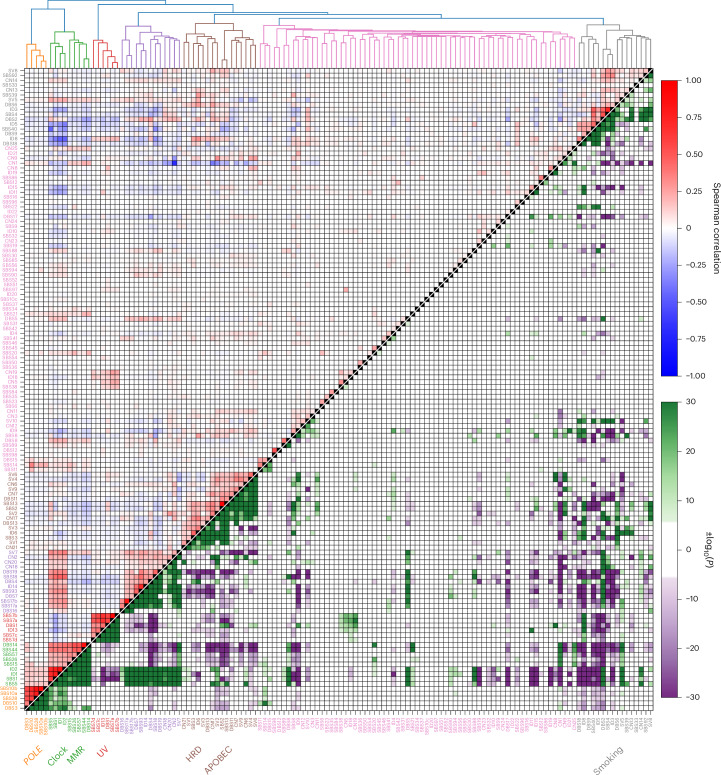
Relationship between SBS, DBS, ID, CN and SV signatures across cancers. Hierarchical clustering (Ward variance minimization with Euclidean distance) of all extracted signatures using log(activity + 1). Distinctly extracted clusters include those related to UV exposure, smoking, HRD, *POLE* mutation and dMMR. The upper triangle shows the Spearman correlation between log(activity + 1) for signatures across all samples, with red squares showing correlated and blue anticorrelated signatures. The lower triangle shows the log *P* value of a two-sided Fisher exact test on the signature having nonzero activity (or, where more than half of the samples have nonzero activity, if the signature activity is above the median across all samples for that signature). Only those with Bonferroni-adjusted *P* < 0.05 are shown with blue and red colors corresponding to negative and positive associations, respectively.

DBS13, composed primarily of mutations to TC-dinucleotides and associated with homologous recombination deficiency (HRD) signatures SBS3, ID6 and CN17 (Spearman *P* = 7.5 × 10^−79^, 5.4 × 10^−96^, 5.3 × 10^−119^; Fig. 3), was identified in breast, ovarian and uterine cancers. The profiles of DBS3, DBS10, DBS12, DBS14 and DBS15 are suggestive of read misalignment (Supplementary Note 1). DBS16 (CA > AC and TA > AT) implies short inversion as a functional basis. DBS15 was observed in central nervous system (CNS), uterine and hepatopancreatobiliary cancers. The mutations primarily contributing to DBS18, NC > AT, are also the basis of SBS8 and SBS22, suggesting that they have a common etiology. While DBS12–DBS16 have cos(sim) > 0.8 with signatures extracted as mentioned in ref. ^13^, DBS17, DBS18 and DBS19 remain new and cannot be constructed from other signatures.

ID19 (5 base pair (bp) insertions), ID20 (C insertions) and ID22 (3–4-bp insertions) varied in rarity, with ID19 common in hematological malignancies and sarcomas. ID21 uniquely featured 2–4-bp deletions. CN25, frequent in CNS, sarcoma and prostate cancers, was characterized by <1-Mb loss of heterozygosity (LOH) deletions, indicative of chromothripsis. However, unlike other chromothripsis-associated CN signatures (CN4–CN8), CN25 is composed of low total CN states with single copy LOH and diploid status.

### Signature relationships

**Extended Data Fig. 2:**
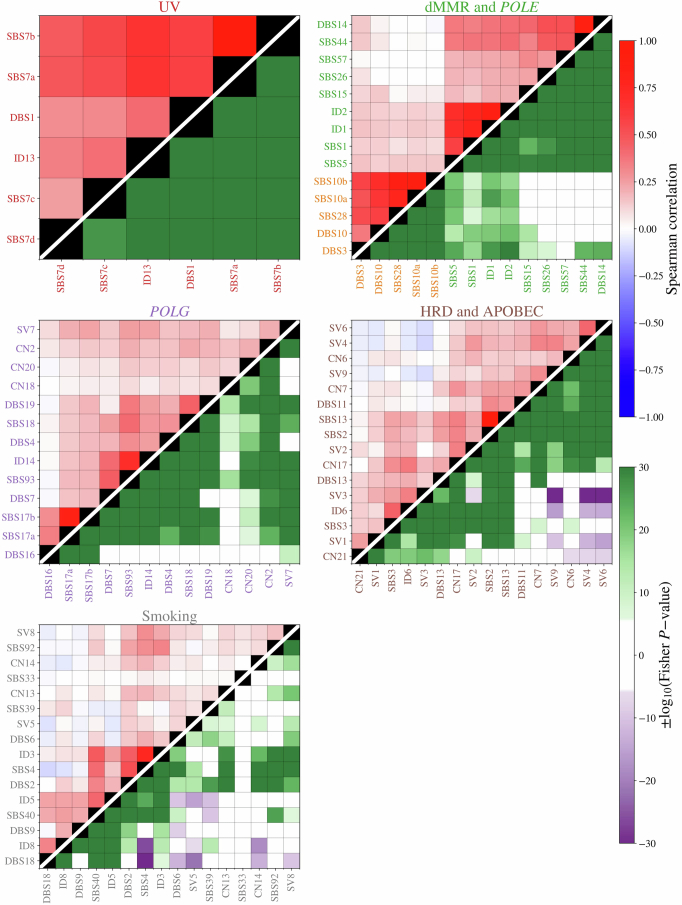
Clusters selected from Fig. 3. Here we have selected 5 clusters from Fig. 3 to view the correlations between activities of different signatures and significance of association between nonzero samples.

Signature clusters linked to ultraviolet (UV) exposure, smoking, Apolipoprotein-B mRNA Editing Catalytic Polypeptide-like (APOBEC) activity, deficient DNA mismatch repair (dMMR), HRD and polymerase epsilon (*POLE*) inactivation^4,6,7,14–16^, as well as clock-like signatures (SBS1 and SBS5), were detected across tumor types (Fig. 3 and Extended Data Fig. 2). We identified clustering of SV4, SV6, SV9, CN6 and CN7, linked to chromothripsis and a new cluster based on SBS93, DBS4, DBS7, ID14, SBS18, DBS19, SBS17a and SBS17b.

**Extended Data Fig. 3:**
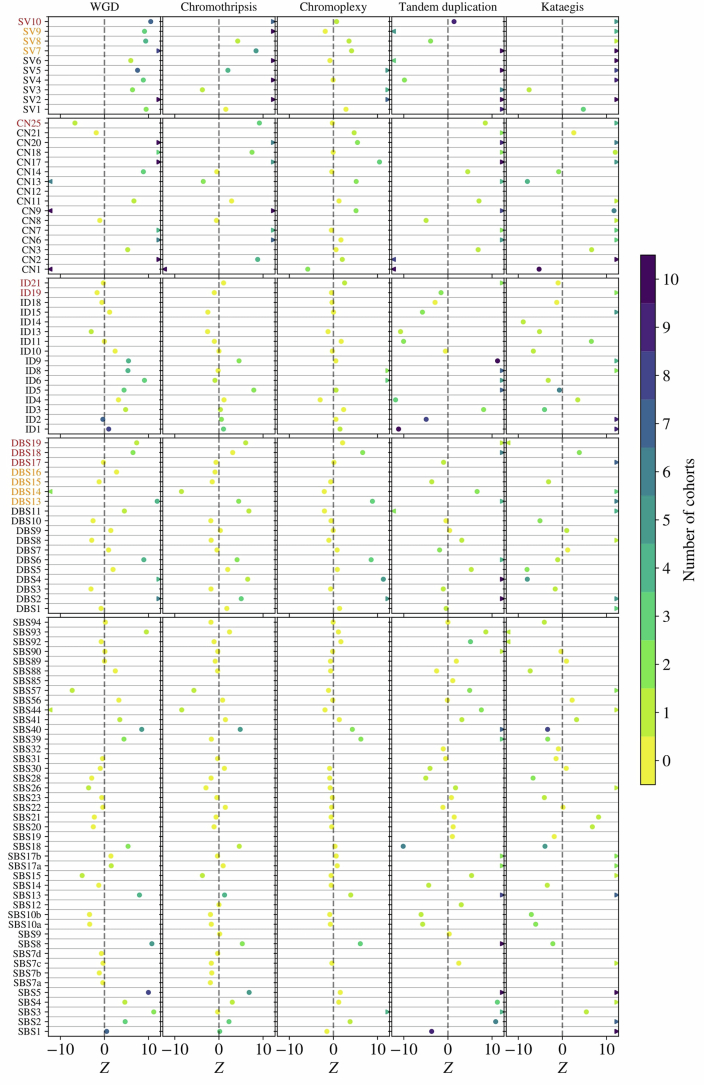
Pancancer associations between signatures and genomic alterations. The associations between genomic alteration events WGD, chromothripsis, chromoplexy, tandem duplications and kataegis are combined pancancer. This is achieved by taking the inverse variance weighted average of association coefficients and renormalizing by the root-sum of inverse variances to estimate the pancancer z-score. The figure shows the pancancer z-scores for each signature against each genomic alteration colored by the number of tumor types cohorts where the association is significant in the same direction as the overall z-score.

SV4, SV6, SV9, CN6 and CN7 were significantly associated with chromothripsis across multiple tumor groups (for example, SV4 with both breast ductal carcinoma (Breast-DuctalCA) and colorectal adenocarcinoma (ColoRect-AdenoCA), *P* = 3.5 × 10^−63^, 2.8 × 10^−36^, *β* = 1.34, 2.26). Additionally, CN6 and CN7 were frequent in tumors displaying whole-genome duplication (WGD; for example, Breast-DuctalCA, *P* = 2.6 × 10^−22^, 1.7 × 10^−48^, *β* = 1.4, 1.9) while CN9 was primarily a feature of non-WGD cancers (*P* = 8.7 × 10^−41^, *β* = −1.6)^17^. WGD is known to induce chromosomal instability (CIN) and was associated with HRD signatures (for example, signatures SBS3, ID6 and CN17 in uterus adenocarcinoma (Uterus-AdenoCA), *P* = 1.0 × 10^−19^, 9.0 × 10^−10^, 2.0 × 10^−15^, *β* = 2.0, 2.6, 4.3). The relationship between signatures with WGD, chromothripsis, chromoplexy, tandem duplications and kataegis is shown in Extended Data Fig. 3 and Supplementary Table 9. WGD was inversely associated with the dMMR signature SBS44, consistent with either being sufficient to influence cancer development (for example, ColoRect-AdenoCA, *P* = 1.3 × 10^−35^, *β* = −2.6)^18^. DBS18 was also associated with WGD and tandem duplications across several cancers (for example, chondrosarcoma (Sarcoma-Chondro), *P* = 5.2 × 10^−4^, 1.2 × 10^−8^, *β* = 1.2, 0.04). Kataegis was associated with SBS2 and SBS13 (for example, Ovary-AdenoCA, *P* = 1.3 × 10^−93^, 5.3 × 10^−76^, *β* = 0.06, 0.06)^6,19,20^ and several SV signature activities (for example, SV2, lung adenocarcinoma (Lung-AdenoCA), *P* = 7.7 × 10^−91^, *β* = 0.04)^21^. However, SV1 and SV3, which are strongly related to HRD, did not show an association with kataegis.

**Extended Data Fig. 4:**
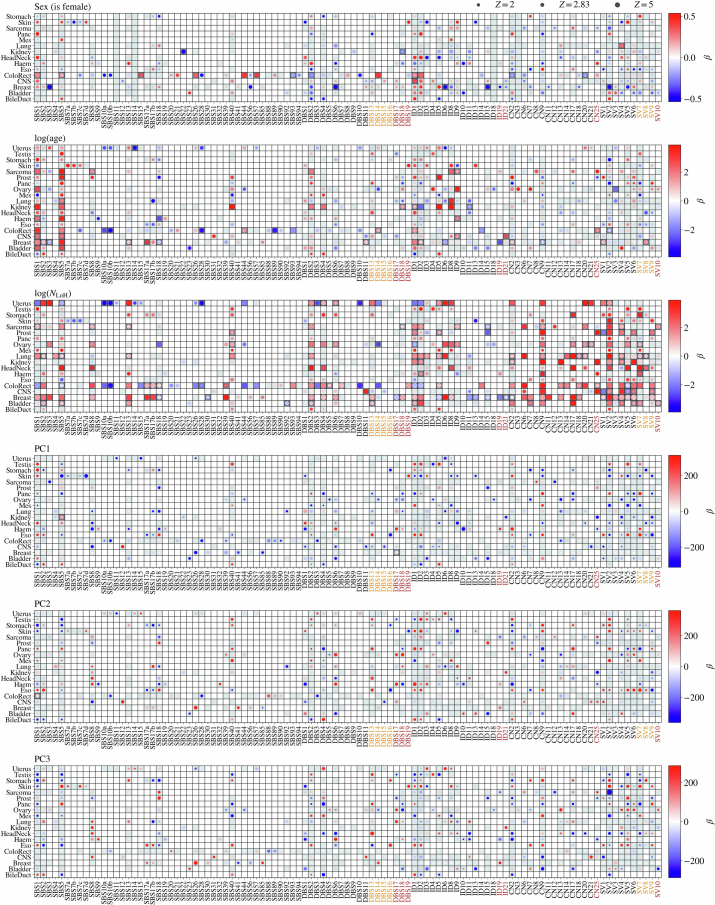
Relationship between signatures and participant sex and age. Signature activities converted to binary values (presence/absence or above/below median) and modeled against five covariates using logistic regression. The association coefficient (rate of change of log odds) for each covariate, signature and tumor type is shown, with the coefficient having been corrected for input parameter normalization. The fits are only performed for tumor types with at least five samples with nonzero signature activity. Point sizes correspond to z-scores of the log-odds and dark gray grid squares have study-wide significant associations under a chi-square test, which is equivalent to |z | > 3.42.

As expected, SBS1 and SBS5 were associated with age at diagnosis in most cancers (Extended Data Fig. 4). SBS88, SBS89 and SBS93 were also enriched in younger ColoRect-AdenoCA patients. SBS88 is caused by *Escherichia* *coli* colibactin exposure and SBS89 appears to be most active in the early phase of life^22^. This raises the possibility that the recent increase in incidence of early-onset colorectal cancer (CRC) may be a consequence of genotoxic microbial exposure^23^. dMMR signature activity (SBS15, SBS26, SBS44) was higher in female ColoRect-AdenoCA patients^24^. ID8 was also associated with age at diagnosis in kidney, lung cancers and sarcomas (*z* = 10.9, 4.8, 5.5; two-sided normal test, *P* = 1.4 × 10^−27^, 1.4 × 10^−6^, 2.9 × 10^−8^). No significant associations were found between any signature and the principal components of germline variation, possibly due to the cohort’s limited ethnic diversity.

**Fig. 4:**
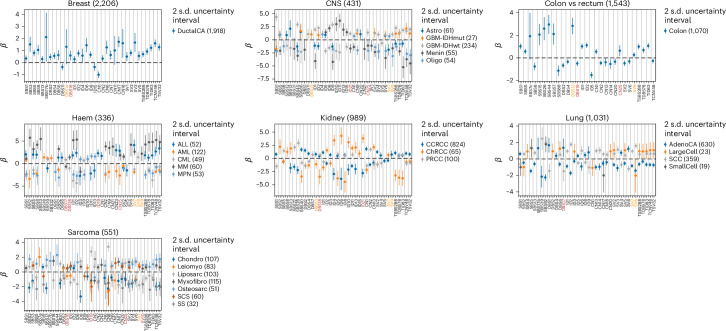
Association between signature activity and tumor histology within each cancer type. The logistic regression coefficient of the signatures being active (or having activity greater than the median) for a tumor having the given histology compared with the remainder of the cohort. Error bars show the 95% confidence interval (2 s.d.) for the regression coefficient and cohort descriptions with abbreviations are listed in Supplementary Table 2. Associations for which Wilks *P* < 0.05 are shown.

Histology-specific associations (Supplementary Table 10 and Fig. 4) included HRD signatures (SBS3, ID6, CN17; Wilks, *P* = 2.4 × 10^−9^, 1.7 × 10^−9^, 1.1 × 10^−22^; logistic, *β* = 1.5, 1.4, 1.7)^25^ and ID8 (*P* = 7.1 × 10^−7^, *β* = 0.7) being significantly more prevalent in Breast-DuctalCA than Breast-LobularCA, indicative of defective nonhomologous end joining (NHEJ). Chromophobe renal cancers (Kidney-ChRCC) were enriched for ID6 (*P* = 1.0 × 10^−9^, *β* = 4.3) and ID21 (*P* = 3.5 × 10^−19^, *β* = 3.8), whereas papillary renal cancers (Kidney-PRCC) were enriched for SBS22 (*P* = 3.7 × 10^−10^, *β* = 2.1), which is linked to aristolochic acid exposure^26^. Osteosarcomas (Sarcoma-Osteosarc) were associated with high activity of APOBEC signatures (SBS2, SBS13, *P* = 2.0 × 10^−7^, 7.0 × 10^−7^, *β* = 1.7, 1.6), leiomyosarcomas (Sarcoma-Leiomyo) with the UV signature SBS7a (*P* = 2.3 × 10^−3^, *β* = 2.0) and liposarcomas (Sarcoma-Liposarc) with the chromothripsis-associated amplification signatures, CN8, SV4 and SV6 (ref. ^27^; *P* = 6.3 × 10^−30^, 5.7 × 10^−9^, 5.3 × 10^−11^, *β* = 3.4, 1.4, 1.5). The observation of SBS7a, SBS7b, SBS7c and DBS1 in some sarcomas may result from the misclassification of some metastatic melanomas^28,29^; however, further work is likely needed to fully elucidate the etiology of these signatures.

Colonic cancers showed evidence of dMMR (SBS15, SBS26, SBS44; *P* = 4.1 × 10^−9^, 3.0 × 10^−14^, 5.6 × 10^−29^, *β* = 2.1, 2.6, 3.0)^30^, whereas rectal cancers tended to feature SBS88, indicative of colibactin exposure as a consequence of pks^+^ *E. coli* infection^31^ (*P* = 7.4 × 10^−7^, *β* = −1.1). Clock-like signature SBS1 activity was higher in IDH wild-type glioblastomas (CNS–GBM–IDHwt) compared to mutated glioblastomas (CNS–GBM–IDHmut), possibly reflecting the older age at diagnosis (*t* test *P* = 5.5 × 10^−27^). In lung, squamous cell carcinomas (Lung-SCC) were enriched for APOBEC signatures (SBS2, SBS13; *P* = 1.7 × 10^−5^, 7.1 × 10^−8^, *β* = 0.6, 0.7). Lung-AdenoCA did not typically feature the smoking signature SBS92 (refs. ^32,33^; *P* = 1.4 × 10^−36^, *β* = −2.3). Among hematological malignancies, multiple myeloma (Heme-MM) and acute lymphocytic leukemia (Heme-ALL) exhibited the highest mutation rates with APOBEC signatures particularly active in Heme-MM (SBS2, SBS13, *P* = 1.6 × 10^−23^, 1.1 × 10^−12^, *β* = 5.8, 5.5), likely to reflect higher APOBEC3G activity^34^.

**Fig. 5:**
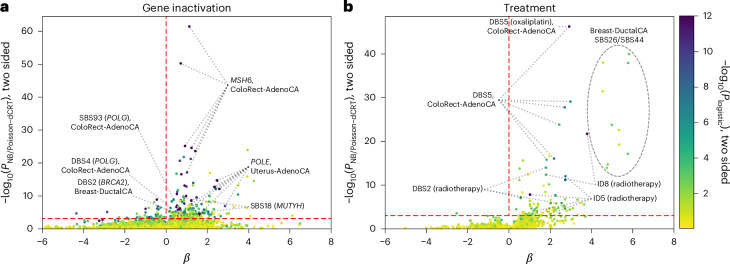
Relationship between DNA repair gene inactivation or treatment exposure and mutational signatures. Associations are computed with the gene knockout parameter for each combination of signature, gene and tumor type, using both an NB and logistic regression linear models. The NB *P* values are computed using conditional resampling and are shown on the *y* axis. The logistic regression *P* values are computed using a Wilks’ likelihood-ratio test and are shown as point colors. **a**, The majority of significant signature–gene inactivation results have positive association coefficients, notably *MSH6* inactivation in CRC and *POLE* gene inactivation in uterine cancer. The horizontal dashed line corresponds to an FDR of 0.01. **b**, Treatment exposures associated with signature activity.

Signature relationships with DNA repair gene mutations and treatments were identified (Fig. 5 and Supplementary Tables 6 and 7; ‘Associations between signature activities and therapy exposure’ and ‘Associations between signature activities and DNA repair gene inactivation’). These include dMMR (SBS44) activity with mutator S homolog 6 (*MSH6)* inactivation in ColoRect-AdenoCA (*P* value, *P* = 3.1 × 10^−61^; effect size, ES = 1.1), *POLE* signatures (for example, SBS10a) with *POLE* inactivation in Uterus-AdenoCA (*P* = 5.8 × 10^−15^, ES = 2.5), HRD signatures (for example, ID6) with germline *BRCA2* mutation in Breast-DuctalCA and Lung-AdenoCA (*P* = 1.0 × 10^−16^, 2.3 × 10^−7^, ES = 1.2, 1.9) and DBS5 with oxaliplatin therapy in ColoRect-AdenoCA (*P* = 1.5 × 10^−56^, ES = 2.9). SBS18 was associated with germline *MUTYH* mutation across several tumors, including ColoRect-AdenoCA, prostate adenocarcinoma (Prost-AdenoCA) and ovarian adenocarcinoma (Ovary-AdenoCA; *P* = 3.3 × 10^−25^, 5.6 × 10^−10^, 9.0 × 10^−9^, ES = 1.0, 2.9, 2.8)^35^. The profile of SBS18 is similar to that of SBS36, which is linked to defective base excision repair and the *MUTYH* mutation^35–37^. Associations between SBS30 activity and *NTHL1* and *FEN1* germline mutations in Breast-DuctalCA confirm a relationship with base excision repair (*P* = 6.2 × 10^−5^, 5.8 × 10^−8^, ES = 2.3, 3.6)^16^.

New associations included CN25 with somatic *MSH6* mutations in ColoRect-AdenoCA, concordant with dMMR (*P* = 2.5 × 10^−15^, ES = 1.4). SBS93 and DBS4, which have correlated activities, were associated with *POLG* inactivation in ColoRect-AdenoCA (*P* = 1.5 × 10^−8^, 2.1 × 10^−6^, ES = 0.4, 0.2) primarily driven by LOH. Contrary to some reports, we found no relationship between SBS17a/SBS17b and 5-fluorouracil (5-FU) exposure^38^. However, the etiology of these signatures is diverse, and a unifying biological basis is yet to be elucidated^39^. SBS7c activity in ColoRect-AdenoCA was linked to *PMS2* mutation (*P* = 7.5 × 10^−10^, *β* = 2.1), hinting at its potential to capture dMMR mutations. ID8, a signature of NHEJ^40–42^, was associated with radiotherapy in all CNS–GBM–IDHwt and Head and Neck squamous cell carcinoma (HeadNeck-SCC; *P* = 8.5 × 10^−15^, 6.9 × 10^−13^, ES = 2.7, 2.0) and in primary cases (*P* = 6.7 × 10^−3^, 1.5 × 10^−13^, ES = 2.4, 2.0). ID5 was also associated with radiotherapy in CNS–GBM–IDHwt and ColoRect-AdenoCA (*P* = 1.3 × 10^−11^, 1.2 × 10^−8^, ES = 2.4, 1.3). Studies have shown *γ* radiation induces microhomologous deletions, such as those in ID8, and A/T deletions in ID5 (ref. ^37^). DBS6 was associated with *BARD1* germline mutations in HeadNeck-SCC (*P* = 4.0 × 10^−9^, ES = 3.7), which suggests a relationship with HRD. SBS39, DBS6 and DBS13 were associated with *BRCA2* somatic mutations in Breast-DuctalCA (*P* = 2.9 × 10^−5^, 3.9 × 10^−22^, 1.9 × 10^−4^, ES = 1.9, 2.1, 1.0). These observations suggest a possible relationship between SBS39, DBS6 and DBS13 and HRD, driven by *BRCA2* loss.

Contradicting previous proposed etiologies, DBS10 was associated with *POLE* signatures (for example, SBS10a, Spearman *ρ* = 6.4, *P* < 1 × 10^−300^) and with somatic *POLE* mutations in ColoRect-AdenoCA and Uterus-AdenoCA (*P* = 5.8 × 10^−40^, 8.9 × 10^−101^, ES = 5.0, 5.8). In contrast to published work^43^, we also found no relationship between SBS20 and *POLD1* inactivation in ColoRect-AdenoCA (*P* = 0.46). However, SBS23 activity was associated with germline *POLD1* mutation in bladder transitional cell carcinoma (Bladder-TCC; *P* = 1.7 × 10^−5^^1^, *β* = 6.9). In Breast-DuctalCA, 67% of 5-FU-treated patients had at least mono-allelic *MLH1* inactivation compared with 28% of the whole cohort (two-sided Fisher exact text, *P* = 1.2 × 10^−5^). The inactivation of *MLH1* is associated with dMMR signatures, likely explaining the observed association of SBS26/SBS44 with 5-FU and related chemotherapies (Fig. 5b).

**Fig. 6:**
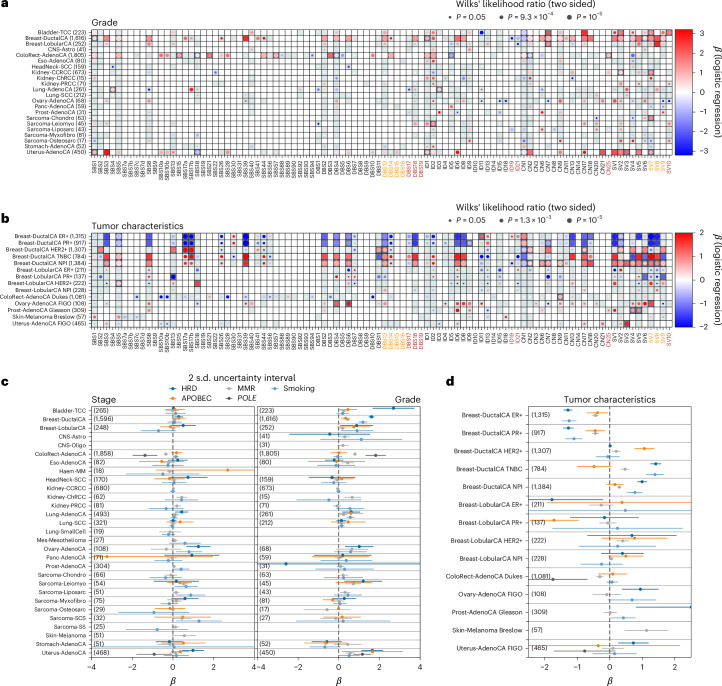
Relationship between mutational signatures and tumor histology. Logistic regression is performed on signature activities (either nonzero or above the median) with tumor clinical properties as covariates within tumor-type groups. **a**, Associations with tumor grade from PHE/NCRAS where the size of the bubble reflects the Wilks *P* value of association and colors show the association coefficient. **b**, As in **a** but for tumor-type-specific features such as hormone status and relevant tumor grades. In **a** and **b**, squares are shaded gray if enough data were available to report an association (minimum of six samples with nonzero activity to control for covariates) and dark gray if the result is study-wide significant across all signature-histology tests (FDR < 0.01). **c**, Signatures with known processes are grouped together and inverse-variance weighted posteriors are shown for the association of tumor TNM stage and grade in each cohort. **d**, As in **c** but for the tumor-type-specific features. Relationships are shown only between signatures and clinical features for which a zero-inflated NB model was successfully fit. This was not always possible, primarily due to sample size (‘Associations between signatures and tumor histologies’).

As reported, HRD signatures were associated with grade (for example, SBS3 and ID6 in Breast-DuctalCA, *P* = 1.4 × 10^−19^, 6.8 × 10^−24^, ES = 1.6, 1.8; Fig. 6a) and were less active in estrogen receptor (ER) and progesterone receptor (PR)-positive Breast-DuctalCA^44^ (for example, SBS3, *P* = 3.4 × 10^−20^, 1.0 × 10^−11^, ES = −1.5, −1.3; Fig. 6b). In contrast, human epidermal growth factor 2 (HER2) status was not associated with HRD (for example, SBS3, *P* = 0.77) and HER2-positive Breast-DuctalCA cancers tended to have higher rates of APOBEC signatures^45,46^ (SBS2, SBS13, *P* = 9.7 × 10^−7^, 6.5 × 10^−7^, ES = 1.0, 1.2; Fig. 6b,d). dMMR (SBS44) signature activity was correlated with higher grade (*P* = 8.8 × 10^−18^, ES = 1.0) but lower stage ColoRect-AdenoCA (*P* = 1.6 × 10^−3^, ES = −0.4; Fig. 6c). Signatures associated with *POLE* inactivation (for example, SBS10a) were associated with high-grade ColoRect-AdenoCA and Uterus-AdenoCA (*P* = 1.5 × 10^−3^, 5.7 × 10^−3^, ES = 1.8, 1.1)^47^. These associations are not study-wide significant due to the small number of samples with nonzero *POLE* signature activity; however, they are clinically important as *POLE*-mutated uterine cancers have a better outlook^48,49^.

### Timing of mutations

**Fig. 7:**
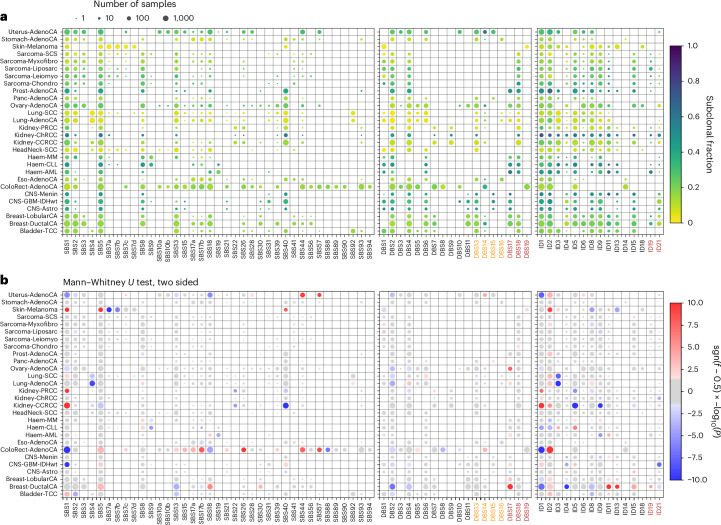
Fraction of mutations that are subclonal. **a**, For each signature in each tumor group, the color shows the fraction of mutations classified as subclonal and the size is the number of samples used to make the estimate. **b**, Significance of the fraction of subclonal mutations for each signature in each tumor group. The colors show whether the mutations associated with a given signature are significantly primarily clonal (blue) or subclonal (red), with the depth of color reflecting the Mann–Whitney *U* test *P* value. Only tumor groups with at least 50 samples and only signatures for which at least 1 sample has >20 subclonal mutations are shown.

**Extended Data Fig. 5:**
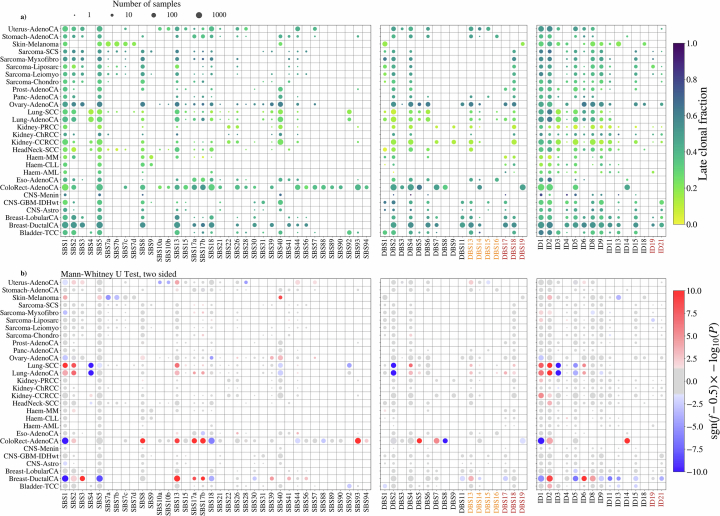
Fraction of clonal mutations classified as late. **a**, The color shows the fraction of late/early clonal mutations which are classified as late by MutationTimeR and the size is the number of samples used to make the estimate. **b**, Significance of ranking of late fraction for all samples with the signature versus all other signatures in the tumor group. The colors show whether the mutations for the given signature are significantly early (blue) in the tumor group or late (red), with the depth of color reflecting the Mann-Whitney U test P-value. Only tumor groups with at least 50 samples and only signatures for which at least one sample has >20 subclonal mutations are shown in the figure.

Timing of mutational signatures was tested by ranked comparison with mutations from other signatures in the same cohort (Fig. 7, and Extended Data Fig. 5 and Supplementary Table 11; Methods—‘Timing of mutations’). Signatures from exogenous processes were significantly more likely to be clonal than those from endogenous processes. This was exemplified by SBS7a and SBS7b in Skin-Melanoma (Wilcoxon rank-sum test, *P* = 1.1 × 10^−14^, 7.7 × 10^−7^), SBS4 in Lung-AdenoCA (*P* = 1.2 × 10^−9^), SBS22 in Kidney-PRCC and clear cell renal cell carcinoma (Kidney-CCRCC, *P* = 9.0 × 10^−6^, 1.3 × 10^−5^) and SBS88 in ColoRect-AdenoCA (*P* = 1.7 × 10^−7^). In contrast, endogenous signature SBS1 (deamination at methylated CpG sites or errors in POL ε proofreading^50^) was significantly more likely to be subclonal than UV mutations in melanomas (Skin-Melanoma, *P* = 1.7 × 10^−15^) or aristolochic acid mutations in Kidney cancers (for example, Kidney-CCRCC, *P* = 2.5 × 10^−31^). Notably, SBS26 and SBS44 were more likely to be subclonal in ColoRect-AdenoCA (*P* = 3.4 × 10^−11^, 2.3 × 10^−8^), suggesting that dMMR mutations frequently arise post-tumourigenesis. SBS2 and SBS13 were predominantly clonal in Bladder-TCC (*P* = 8.9 × 10^−5^, 1.3 × 10^−3^). However, in Breast-DuctalCA, ColoRect-AdenoCA, Lung-AdenoCA and Lung-SCC, clonal APOBEC mutations occur significantly later than other processes, including SBS1, SBS5 and SBS4, which suggest that APOBEC mutations occur in cells before the last clonal sweep (for example, Breast-DuctalCA, SBS2/SBS13, *P* = 7.2 × 10^−3^, 5.6 × 10^−12^). Mutations attributable to SBS18, which has a similar profile to SBS36, were more likely to be clonal than other mutations in ColoRect-AdenoCA and Uterus-AdenoCA (*P* = 3.9 × 10^−5^, 1.7 × 10^−6^). Because SBS18/SBS36 is associated with a germline *MUTYH* mutation, this implies that mutations as a consequence of *MUTYH* inactivation can accumulate before tumourigenesis. As further evidence for this, we found that clonal SBS18 mutations were significantly associated with *MUTYH* germline mutations in ColoRect-AdenoCA, Ovary-AdenoCA and Prost-AdenoCA (*P* = 3.3 × 10^−25^, 9.0 × 10^−9^, 5.6 × 10^−10^, ES = 1.0, 2.8, 2.9; Supplementary Table 6).

SBS7c mutations were more likely to be subclonal than other signatures in ColoRect-AdenoCA (*P* = 3.1 × 10^−3^), which reinforces our hypothesized relationship with dMMR due to association with *PMS2* inactivation. Of the seven signatures (SBS93, DBS4, ID14, DBS7, SBS18, DBS19, SBS17a and SBS17b) clustered together in Fig. 3, SBS93, ID14, DBS7, SBS17a and SBS17b occur late in ColoRect-AdenoCA (*P* = 4.2 × 10^−22^, 5.9 × 10^−21^, 4.0 × 10^−6^, 3.9 × 10^−23^, 2.4 × 10^−49^) and levels of SBS93, DBS4 and ID14 are associated with *POLG* inactivation (*P* = 1.5 × 10^−8^, 2.1 × 10^−6^, 1.3 × 10^−3^), as well as nonzero activity of DBS7, SBS17a and SBS17b (*P* = 2.2 × 10^−6^, 5.2 × 10−4, 2.7 × 10^−5^), while no significant associations were found for SBS18 and DBS19. DBS17 was highly subclonal, particularly in Breast-DuctalCA and Ovary-AdenoCA (*P* = 1.8 × 10^−18^, 1.5 × 10^−8^), suggesting that this signature may be driven by endogenous processes late in tumor development. ID21-related mutations were relatively clonal across multiple cohorts (for example, Kidney-ChRCC, *P* = 7.6 × 10^−5^), suggesting they are caused by exogenous processes or mutagenesis in normal tissue. This was also the case for ID9 (for example, Kidney-CCRCC, *P* = 6.4 × 10^−17^), which has been reported^7^ but has unknown etiology.

**Extended Data Fig. 6:**
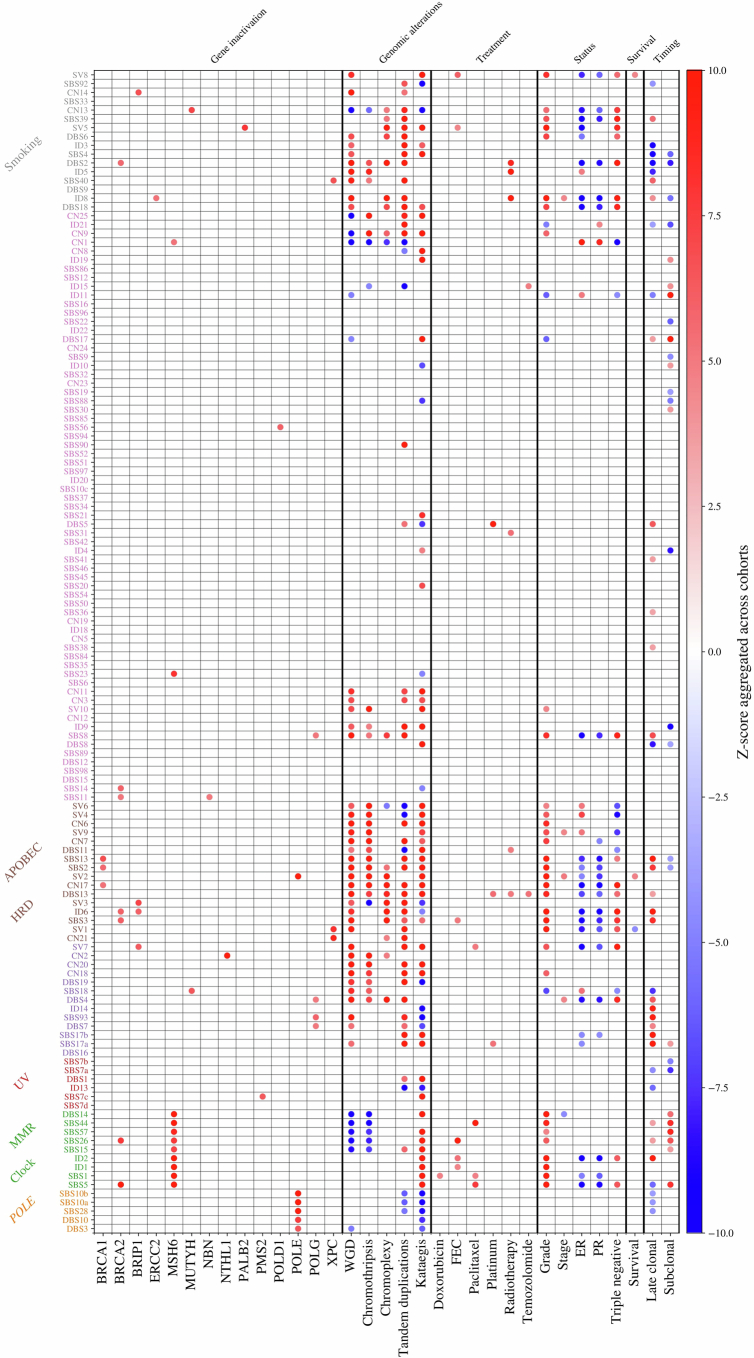
Aggregated signature associations. All association results from gene inactivations, genomic alterations, treatment exposures, clinical status, overall survival and mutation timing across tumor types. The colors are the aggregated z-scores across cohorts as described in ‘Aggregated signatures’.

Our results are summarized in Extended Data Fig. 6, where we combined the relationships between signatures and multiple lines of evidence aggregated across all cancers, providing insight into the etiologies and interrelationships for all 134 signatures (Supplementary Table 3).

### Clinical relevance of signatures

**Fig. 8:**
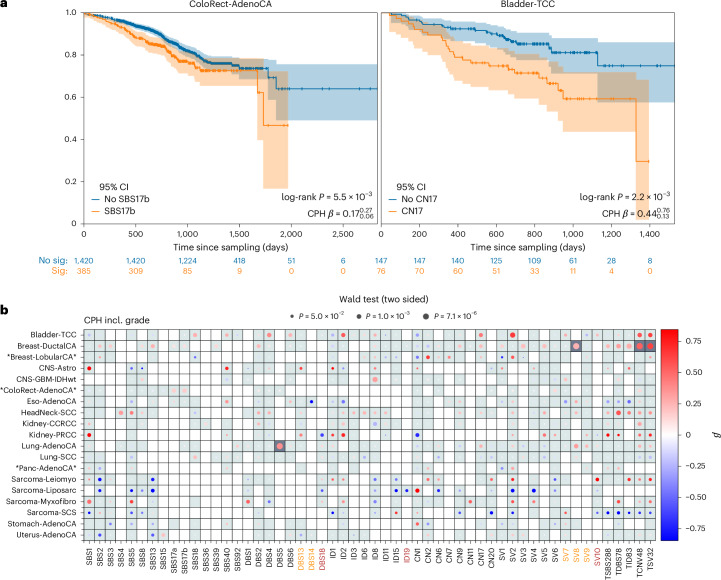
Relationship between mutational signatures and patient OS. **a**, Kaplan–Meier survival curves for ColoRect-AdenoCA and Bladder-TCC stratified by SBS17b and CN17 activity. **b**, CPH tests of OS adjusting for patient age, sex, germline principal components and tumor grade. Dark gray grid squares show study-wide significant associations against all survival tests with FDR < 0.01 (ref. ^63^). TSBS288, TDBS78, TID83, TCNV48 and TSV32 correspond to the total mutation counts of the five signature types explored in this work. Positive association corresponds to reduced survival. Only tumor groups and signatures with at least one association at *P* < 0.1 are shown. Any cohorts with significant PH or LB test results across signatures are marked with an asterisk. This is determined by comparing the number of associations in the cohort with PH or LB, *P* < 0.01, with the total number of association tests, assuming a binomial distribution with 1% expected probability. We mark signatures with an asterisk if the survival probability of the binomial test is <0.05/number of signatures. LB, Ljung–Box; CI, confidence interval.

Concurrent with the development of methods for mutational signature identification has been the recognition that signatures can complement driver gene identification in predicting patient prognosis and treatment response^51^. Several signatures were associated with overall survival (OS; Fig. 8 and Supplementary Table 12). Notably, HRD (SBS3) and APOBEC-related signatures (SBS2 and SBS13) in Breast-DuctalCA were associated with a poorer OS after adjusting for tumor grade (two-sided Wald *P* = 2.2 × 10^−2^, 3.5 × 10^−2^, 9.3 × 10^−3^, Cox proportional hazard (CPH) *β* = 0.2, 0.2, 0.3)^52^. However, these no longer remained significant after controlling for ER status. Signature SBS17b was associated with reduced OS in ColoRect-AdenoCA (*P* = 1.9 × 10^−3^, *β* = 0.2). CN17, active in 88 of 296 Bladder-TCC, was significantly associated with reduced OS (*P* = 5.9 × 10^−3^, *β* = 0.49). DBS5 was associated with reduced OS in Lung-AdenoCA (*P* = 4.8 × 10^−6^, *β* = 0.4); however, this observation is likely confounded by indication, as DBS5 can be a consequence of platinum therapy (as shown by its association with oxaliplatin therapy in ColoRect-AdenoCA (*P* = 1.5 × 10^−56^)^37^). ID8 burden, reflective of NHEJ inactivation, was associated with reduced OS in CNS–GBM–IDHwt (*P* = 1.3 × 10^−4^, *β* = 0.3). However, this observation is likely confounded by tumor type (165 primary versus 8 recurrent tumors), with the association attenuated after restricting the analysis to primary CNS–GBM (*P* = 0.073). While these associations are not study-wide significant after adjusting for multiple testing, they provide indications of potential lines of evidence for follow-up studies.

Aside from providing insight into mutagenesis, mutational signatures have potential as biomarkers for identifying cancers arising from radiotherapy and chemotherapy. Notably, they reflect the DNA repair capacity of cancer cells, and as such are being shown to predict response to DNA-damaging or other therapies^53^. For example, HRD signatures provide an indication for PARP inhibition therapy^54^ and sensitivity to platinum chemotherapy^55^. Using nonzero activity in at least two of SBS3, ID6 and CN17 in a tumor as an indicator of HRD, 381 (17%) breast, 134 (30%) ovarian, 41 (4%) lung, 33 (5%) uterus, 28 (5%) sarcomas and 23 other cancers showed evidence of HRD. The etiological basis of HRD was identifiable in 16% breast and 14% ovarian cancer cases on the basis of biallelic inactivation of *BRCA1*, *BRCA2*, *PALB2*, *BRIP1* or *RAD51B* through germline and somatic mutations. Other cases may be caused by promoter methylation; however, these data are not available for 100KGP samples. Of the 55,920 breast and 4,295 ovarian cancer patients diagnosed in the United Kingdom each year^56^, our analysis suggests that 7,784 and 1,088, respectively, may benefit from HRD-targeting therapies, far more than are currently eligible based simply on the identification of breast and ovarian cancer gene mutations.

While high levels of dMMR are primarily a feature of uterine cancers (32%) and CRC (18%), it is also a feature of subsets of other tumors, including those of the lung, ovaries, prostate, kidney and breast. However, only 15% dMMR tumors showed evidence of *MSH6*, *MSH2* or *MLH1* inactivation. The identification of dMMR signatures has therapeutic relevance for solid tumors as it dictates eligibility for checkpoint inhibition^57^, sensitivity to 5-FU^58^ and WRN inhibition^59,60^. In glioma, dMMR signatures highlight tumors that are likely to respond adversely to temozolomide and thiopurine therapy due to mutagenesis of driver genes such as *TP53* (ref. ^53^). Signatures corresponding to nucleotide excision repair patterns associated with *ERCC2* disruption have recently been reported to provide a biomarker for sensitivity to platinum treatment^61^. Finally, APOBEC signatures SBS2 and SBS13 that we identified in 88% bladder, 89% head and neck, 69% breast, 37% lung, 38% sarcoma and overall 29% of the 10,983 tumors have been linked with sensitivity to ATR inhibitors^62^.

## Discussion

This study extracts and analyzes the full complement of SBS, DBS, ID, CN and SV signatures from a single. Twenty-six of the signatures we extracted have not previously been cataloged by COSMIC, including nine that have not been reported in previous studies. Ref. ^13^ has also recently extracted SBS and DBS signatures in 100KGP. A discussion of the differences between that study and ours is provided in Supplementary Note 5.

Using the curated histology data, we compared signature activities across tumor groups and identified signatures associated with processes that are more prevalent in some tumor types than others. For example, SBS22 has previously been linked to exposure to the nephrotoxic aristolochic acid. We now report a relationship between SBS22 and renal cancer, with specificity for the papillary subtype. We also demonstrated associations between signatures with previously unknown etiologies and inactivation of DNA repair genes or exposure to treatments. Notably, the new signature CN25 is associated with *MSH6* inactivation.

By determining the clonal and subclonal fraction of mutations attributed to each SBS, DBS or ID signatures, we found mutational signatures caused by exogenous processes, such as UV light or tobacco smoke exposure, occur earlier in tumourigenesis than endogenous processes such as DNA repair defects, particularly dMMR.

Our study constitutes a comprehensive COSMIC reference for SV signatures. As well as verifying the previously determined etiologies of SV3 and SV5 as HRD-driven^11^, we also hypothesize that SV4, SV6 and SV9 are a result of chromothripsis.

We have examined the relationship between patient outcome and mutational signatures on a large scale. Our analysis shows that signature analysis can complement conventional clinical staging in predicting patient prognosis. Moreover, signatures of HRD, dMMR and APOBEC activity can be used as indicators for patient response to multiple therapies, including immune checkpoint inhibitors.

The methodology we have applied herein to identify new signatures is designed to maximize the linear independence of the signature catalog. New signatures were added to the COSMIC reference list one at a time if they could not be decomposed to the reference list with cos(sim) > 0.8, prioritizing signatures with the smallest maximum cosine similarity to any reference signature. This ensures that any new signature is sufficiently linearly independent of all current signatures. However, this does not guarantee a set of signatures that are linearly independent of one another. If a signature already included in the reference list could be composed of two new signatures to be added, the resulting signatures would be linearly dependent. This issue is already present in COSMIC, where previously added signatures may be composed of later additions to the list. A total of 66% (42/64) COSMIC SBS signatures can be produced by linear combinations of other COSMIC SBS signatures with cos(sim) > 0.8, as shown in Supplementary Table 3. Only one of the new signatures within this work (DBS18) can be composed of other signatures (DBS9 and DBS19, cos(sim) = 0.85). Hence, we believe that a new approach to producing signature reference catalogs, which can be updated over time, is required to allow for robust signature extraction and deconvolution. This is especially relevant if signature analysis is to become integrated into routine clinical care, to address statistical issues surrounding assignment of signatures and potential errors from sequencing artifacts and downstream analyses (Supplementary Note 6).

## Methods

### The 100KGP cohort

Ethics approval was provided to the 100KGP by the HRA Committee East of England–Cambridge South Research Ethics Committee (REC reference 14/EE/1112). Written informed consent was provided by all patients recruited to the study (patient consent form available at https://www.genomicsengland.co.uk/assets/forms/Participant-consent-form-for-patients-with-a-rare-genetic-disease-and-their-adult-relatives-R1.pdf). Study oversight was subsequently conducted by Genomics England through regular reporting updates to the GeCIP steering committee and data Airlock committee. The analyzed cohort (100KGP, release v11) comprised tumor–normal (T/N) sample pairs recruited to 100KGP through 13 Genomic Medicine Centers (GMCs) across England. We restricted our analysis to samples with high-quality data from PCR-free fresh-frozen material and samples that could be assigned to a specific tumor histology. This yielded 10,983 samples from 10,975 participants (41 tumor histologies, across 16 tissue types). Comprehensive clinico-pathology information on the patients is provided in Supplementary Table 2, including the size of each individual cohort, the number of male and female participants and the age demographic. Correlations between genomic features were identified using logistic, linear and negative binomial (NB) regression and Fisher’s exact test (‘Associations between signature activities and therapy exposure’ to ‘Signature and gene inactivation mock tests’).

### WGS and mutational analyses

Illumina conducted WGS of paired T/N DNAs. We corrected for reference bias in variant calling using FixVAF^64^. Variant Call Formats (VCFs) were annotated using Ensembl Variant Effect Prediction^65^. Strelka was used to call somatic variants^66^, and a four-stage pipeline incorporating Battenberg^67^ for CN calling and a consensus approach based on Manta^68^, Lumpy^69^ and Delly^70^ for calling somatic structural variants (SVs). De novo extraction of mutational signatures, including decomposition to known COSMIC signatures^71^ (v3.3), was performed using SPE^10^. The relative evolutionary timings of candidate driver mutations were calculated through MutationTimeR^72^. Complete details on sample curation, tumor purity estimation, WGS, somatic variant calling, mutation annotation, CN alteration (CNA) calling/annotation, somatic SV calling/annotation, WGD, as well as the identification of kataegis and chromothripsis are provided in ‘100KGP WGS data’ to ‘Selection of study samples’.

### Statistics and reproducibility

The detailed information on statistical tests used for associations between signature activities and the different biological and clinical factors considered in this study is presented in ‘Associations between signature activities and therapy exposure’ to ‘Aggregated signatures’. For associations among signature activities and genomic alterations, therapy exposure and tumor suppressor gene inactivation, we used distilled conditional randomization test (dCRT)^73,74^ that drastically reduces false-positive rates compared with a Wilks’ likelihood-ratio test. A total of 3,692 genomic alterations associations were tested (*P* < 3.9 × 10^−3^, false discovery rate (FDR) = 0.01, Benjamini–Hochberg^63^), 2,062 treatment exposure associations (*P* < 3.9 × 10^−3^, FDR = 0.01), 39,696 gene inactivation associations (*P* < 2.5 × 10^−4^ for FDR = 0.01) and 2,299 clinical status associations (for example, grade, stage and hormone receptor statuses, *P* < 1.3 × 10^−3^, FDR = 0.01). A total of 1,180 associations were tested between signature presence and tumor histology with Wilks’ likelihood-ratio tests on logistic regression models (*P* < 3.1 × 10^−3^, FDR = 0.01). A total of 1,141 tests for the significance of signature mutation timing were performed with Wilcoxon rank-sum tests (*P* < 1.2 × 10^−3^, FDR = 0.01). A total of 776 tests were performed to assess the association between signature activity and OS survival. These were tested using a Wald test of the association coefficient for the signatures in a CPH model (*P* < 7.1 × 10^−6^, FDR < 0.01).

This study used measurements recorded as part of the 100KGP, for which we had no control over the experiments performed. As such, no statistical method was used to predetermine sample size, the experiments were not randomized and the investigators were not blinded to allocation during experiments and outcome assessment.

### 100KGP WGS data

A total of 14,129 paired tumor-germline samples were obtained from V11 of the 100KGP^75^. Samples had been prepared using Illumina TruSeq DNA PCR-free library preparation kit and sequencing was performed on a HiSeq X, producing 150-bp paired-end reads to 33× depth for germline and 100× for tumor samples. The 100KGP program excludes outliers with poor sequencing quality (based on the percentage of mapped reads, percentage of chimeric DNA fragments, average insert size, AT/CG dropout and unevenness of local coverage). Alignment was performed to the *Homo sapiens* GRCh38decoy assembly using Isaac (v03.16.02.19)^76^.

### Collection and processing of clinical data

Clinical and demographic data were obtained from NHS Digital (NHSD), Public Health England’s National Cancer Registration and Analysis Service (PHE–NCRAS) and the GMCs through the Genomics England Research Environment^77,78^. Additional data were also obtained from associated histology reports where available. Sequenced tumor samples were matched to their respective PHE–NCRAS records using the tumor sampling date and PHE–NCRAS treatment dates, allowing a maximum discrepancy of 28 days. The data obtained included sex, year of birth, date of cancer diagnosis, date of last clinical follow-up, survival outcome and date of death if applicable; tumor histology; anatomical site of the primary tumor; anatomical site sampled; and whether the sample was taken from a primary tumor, a metastasis or a recurrence of a primary tumor. For some variables, data were obtained from multiple sources (GMC, NHSD, PHE–NCRAS). Potential conflicts between these data sources were manually reviewed. A.J.C., who was not clinically trained, performed the initial review. Conflicts requiring clinical knowledge were subsequently reviewed by R.S.H. or D.N.C., both of whom are clinicians. Sequenced tumors were assigned to 1 of 41 tumor groups depending on the tumor histology and originating tissue (Supplementary Table 2).

Information on whether participants had received systemic treatment or disease-associated radiotherapy before sampling was obtained from NHSD and PHE–NCRAS. Admitted patient care and outpatient records related to systemic treatment were obtained from NHSD tables using Office of Population Censuses and Surveys-4 codes. Records related to systemic treatment were also obtained from the PHE–NCRAS AV treatment and Systemic Anti-Cancer Therapy tables. Records related to radiotherapy were obtained from the PHE–NCRAS AV treatment and National Radiotherapy Dataset tables.

### Calling germline and somatic variants

Calling of germline variants was performed using Starling (v2.4.7)^76^. Calling of somatic single-nucleotide variants (SNVs) and indels was performed using Strelka (v2.4.7)^79^. In addition to the default Strelka filters, somatic variants were removed if they met any of the following criteria:

- Had a population germline allele frequency of ≥1% in 100KGP or gnomAD^80^.
- Had a somatic frequency of ≥5% in 100KGP tumor samples.
- Overlapped a simple repeat as defined by Tandem Repeats Finder^81^.
- Was an SNV likely resulting from systematic mapping or calling artifacts. Likely artifacts were identified by computing the ratio of tumor allele depths at each somatic SNV site and comparing against the ratio of allele depths at the same site in a panel of normal samples, which comprised 7,000 nontumor genomes from the 100KGP cohort. Allele depths at each site were counted in the panel of normal individuals, including only individuals not carrying the relevant alternate allele. To replicate Strelka filters, duplicated reads were removed before counting and mapping quality of ≥5 and base quality of ≥5 thresholds were applied. SNVs with a Fisher’s exact test Phred quality score of ≤50 were excluded.
- Was an indel in a region of high levels of sequencing noise, where ≥10% of the base calls in a window extending 50 bp to either side of the indel call have been filtered out by Strelka.
- Was an indel within 10 bp of an indel called in Genomics England or in gnomAD in >1% germline samples.

### Calling somatic CNAs

Somatic CNAs were called using a five-stage procedure as per ref. ^67^.

#### Stage I: initial CNA profiling

Clonal and subclonal CNAs were profiled using Battenberg^67^. Briefly, alleleCount-FixVAF was used to count reads supporting single-nucleotide polymorphism (SNP) reference and alternate alleles^64^. Heterozygous SNPs were then phased with SHAPEIT2 (v2.r904)^82^. Piecewise constant fitting was used to segment-phased SNPs^83^ and CNAs with evidence of subclonality were identified using *t* tests. Sample tumor purity and ploidy were estimated using the approach described in ref. ^84^. Sequencing data were aligned to hg38 and it was therefore necessary to convert SNP positions to hg37 before phasing and convert output segments back to hg38.

#### Stage II: using variant allele frequency (VAF) distributions to evaluate profile concordance

Factors influencing expected VAFs include (1) the fraction of tumor cells containing the variant, (2) CNAs at the variant site, (3) the number of chromosome copies carrying the variant (multiplicity) and (4) tumor sample purity^85^. Given the tumor CN profile and sample tumor purity, we can expect to observe enrichment of variants with VAFs approximating specific values, representing clonal variants present in all tumor cells^84^. Failure to observe this enrichment indicates that either the CN profile or tumor sample purity is incorrect. We therefore used SNV VAF distributions to assess the CNA profiles and sample tumor purities computed by Battenberg^86^.

Autosomal genome segments with CN states of 1:1, 1:0, 2:2, 2:1, 2:0 with no evidence of subclonal CNAs were considered when assessing SNV VAF distributions. The five CN states were considered separately as expected clonal SNV VAFs and possible variant multiplicities differ between states^84^. A CN state was not considered if it corresponded to genome regions containing <5% of all SNVs. Expected VAF distribution peak locations were computed as:

$$\frac{{\rho }_{\mathrm{Battenberg}}m}{2\left(1-{\rho }_{\mathrm{Battenberg}}\right)+{\rho }_{\mathrm{Battenberg}}{\psi }_{v}}$$

where *ρ*_Battenberg_ is the sample tumor purity output by Battenberg, *ψ*_*v*_ is the tumor ploidy at the variant site and *m* is the variant multiplicity (which can equal 1 or 2 in 2:2, 2:1 and 2:0 states and only 1 in 1:1 and 1:0 states). VAF distribution peaks were identified using kernel density estimation implemented in the peakPick R package (v0.11)^87^. Peaks with densities of <0.3 were excluded. For each CN state, the expected peak location corresponding to the greatest variant multiplicity was matched to the observed VAF distribution peak with the greatest VAF. Remaining expected peak locations were then matched to the observed peaks with the most similar VAFs. Tumor heterogeneity can inhibit VAF peak detection, and therefore, for samples where ≥1 expected peaks were considered, the expected peak furthest from the respective matched observed peak (in terms of VAF) was discarded. Sample tumor purity (*ρ*_*i*_) was then estimated for each remaining expected peak using its matched observed peak VAF:

$${\rho }_{i}=\frac{2a}{m+\omega \left(2-{\psi }_{s}\right)}$$

where *ω* is the VAF of the matched observed peak and *ψ*_*s*_ is the state ploidy. A single new purity estimate (*ρ*_new_) was then computed as a weighted average of the peak-wise purity estimates:

$${\rho }_{\mathrm{new}}=\mathop{\sum }\limits_{i}\frac{{{n}_{i}\rho }_{i}}{{n}_{\mathrm{all}}{q}_{i}}$$

where *q*_*i*_ is the number of considered variant multiplicities for the CN state, *n*_*i*_ is the number of SNVs in genome regions with the CN state and *n*_all_ is the number of SNVs in genome regions of all considered CN states. Finally, the difference between the Battenberg purity estimate and the peak-wise purity estimates was used to assess CNA profile quality:

$$\eta =\mathop{\sum }\limits_{i}\frac{{n}_{i}\left|{\rho }_{{\rm{i}}}-{\rho }_{\mathrm{Battenberg}}\right|}{{n}_{\mathrm{all}}{q}_{i}}.$$

#### Stage III: profile quality assessment

The following criteria were used to assess CNA profile quality:

- SNV VAF distribution peaks found at expected locations (defined as *η* < 5%).
- A clonal variant cluster was identified by DPClust^67^. This was defined as a variant cluster with a cancer cell fraction (CCF) between 0.9 and 1.1, containing ≥5% of all SNVs.
- No ‘super-clonal’ variant clusters were identified by DPClust. These were defined as variant clusters with CCFs > 1.1, containing ≥5% of all SNVs.
- If most of the genomes are categorized as 2:2 (tetraploid), then an SNV VAF distribution peak in 2:2 regions corresponding to a variant multiplicity of 1 was observed.
- No homozygous deletions of >10 Mb are called.

CNA profiles not satisfying at least one criterion were deemed to fail and were reprofiled (that is, proceeded to stage IV). CNA profiles satisfying all criteria were deemed to pass and were used in subsequent analyses.

#### Stage IV: CNA reprofiling

Samples failing the CNA profile quality assessment were reprofiled a maximum of three times using alternative tumor sample purity and ploidy estimates. CNA profiles that still failed quality assessment after three attempts were excluded from subsequent analyses. New purities (*ρ*_new_) were iteratively re-estimated as per stage II, while new ploidies (*ψ*_new_) were estimated as per ref. ^84^:

$${\psi }_{\mathrm{new}}=\frac{{\rho }_{\mathrm{Battenberg}}\left({\psi }_{\mathrm{Battenberg}}-2\right)+2{\rho }_{\mathrm{new}}}{{\rho }_{\mathrm{new}}}$$

#### Stage V: manual review

Three tumor groups (Heme-MPN, Ovary-AdenoCA and Testis-GCT) had higher than expected sample proportions failing CNA profile quality assessment. High failure rates in Heme-MPN and Testis-GCT were due to low SNV, which complicated the automatic identification of SNV VAF distribution peaks. Conversely, the high failure rate in Ovary-AdenoCA was primarily due to the presence of large homozygous deletions. For these three tumor groups, we therefore manually reviewed CNA profiles failing quality assessment and passed them when appropriate. The biological plausibility of large homozygous deletions in Ovary-AdenoCA tumors was assessed by considering the involvement of genes classified as essential in ovarian cancer cell lines in DepMap^88^. If a homozygous deletion contained no essential genes, then it was considered biologically plausible, and the CNA profile was considered passable.

### Calling and classifying somatic SVs

Somatic SVs were called using Delly^70^, Lumpy^69^ and Manta^68^ with a graph-based consensus approach, with support from CNA profiles. SVs were first called using the three SV callers, with default parameters. Delly was run with postfiltering of somatic SVs using all normal samples. SVs from the individual callers were removed if (1) any reads supporting the variant were identified in the matched normal sample, (2) <2% tumor reads supported the variant, (3) either variant breakpoint was located on a nonstandard reference contig (not chromosomes 1–22, X or Y), or (4) either variant breakpoint was located in a centromeric or telomeric region. Remaining SVs were merged with a modified version of PCAWG Merge SV, allowing 400-bp slop at the breakpoint positions^12^. SVs were included in the final SV dataset if they were supported by at least two of the three SVs callers, or by only one SV caller but with a breakpoint of <3 kb from a CNA segment boundary.

xTea was used to call somatically acquired long interspersed nuclear element (LINE-1) retrotransposition events^89^. Alu elements, SINE-VNTR-Alu elements and processed pseudogenes comprise ≤3% cancer retrotransposition events and were therefore not analyzed^90^. Retrotransposition events were not considered in subsequent SV analyses as they are mechanistically distinct from other SV-generating events^90^. SVs were categorized as likely retrotransposition events and excluded if (1) a transduced region was identified in the same tumor sample within 10 kb of either rearrangement breakpoint, or (2) a transduced region was identified within 10 kb of either rearrangement breakpoint in ≥1% tumor samples. A threshold of 10 kb was imposed as the majority of somatically acquired transductions were <10 kb from a LINE-1 element^91^.

Using ClusterSV^20^, rearrangements were grouped into footprints and clusters based on their proximity within the genome, rearrangement size and overall rearrangement number in the genome. Rearrangement footprints represent sets of rearrangement breakpoints that are positionally associated. Rearrangement clusters represent sets of rearrangements that are mechanistically associated and were classified as being a simple (deletions, tandem duplications, balanced inversions, balanced and unbalanced translocations, and simple unclassified events) or complex (chromoplexy, chromothripsis and complex unclassified events) event. Rearrangement clusters comprising ≤2 or ≥3 individual rearrangements were defined as simple and complex events, respectively.

Rearrangement clusters were defined as a chromothripsis event if they met the following criteria:

- At least six interleaved intrachromosomal rearrangements.
- A contiguous series of four genome segments oscillating between two CN states, or five genome segments oscillating between three CN states.
- No evidence that the distribution of intrachromosomal fragment join orientations diverge from a distribution with equal probabilities for the four orientation categories (duplication-like, deletion-like, head-to-head inversion and tail-to-tail inversion) at an FDR of 0.2.

Rearrangement clusters were defined as a chromoplexy event if they met the following criteria:

- Comprises between 3 and 30 rearrangements.
- Contained a chain of rearrangements spanning at least three chromosomes. Chains were defined using a graph-based approach, in which nodes represent breakpoints and are connected by an edge if they fall within 1 Mb of each other and are not involved in the same rearrangement.
- At least 50% rearrangement footprints represent balanced translocations, either with a deletion bridge between the break ends or no observed CN change.

Kataegis identification was performed using SigProfilerClusters. Briefly, SigProfilerClusters calculates a sample-dependent IMD threshold that considers regional differences in mutation rates, variant allele fractions and CCFs of adjacent mutations, and delineates mutations into clustered and nonclustered groups^92^.

### Selection of study samples

Sequenced tumor samples were excluded if clinical data were missing or if unresolvable conflicts existed between the clinical data sources (GMCs, NHSD, PHE–NCRAS, histology reports). In total, 2,251 of 14,129 (15.9%) tumor samples were excluded based on the following criteria:

- Sex reported by NHSD, PHE–NCRAS and/or the GMC did not match the sex inferred from the sequencing data.
- Sample could not be assigned to 1 of the 41 tumor groups, either because of missing or conflicting tumor histology or originating tissue data, or because the malignancy was not represented by one of the groups.
- Missing or conflicting data meant it was unclear whether a primary tumor, a metastasis or a recurrence of a primary tumor was sampled.
- Missing or conflicting data concerning the day of sampling.
- The participant was less than 18 years old on the day of sampling.

Tumor sample purity and sequencing data quality affect the sensitivity and precision of variant calling^66^ and we therefore also excluded samples using the following quality control procedures (Supplementary Table 1). In total, 267 of 11,878 (2.2%) tumor samples with required clinical data available were excluded based on sequencing data using the following criteria:

- If cross-contamination of the tumor sample was >1%, as estimated by VerifyBamID^93^.
- If cross-contamination of the matched germline sample was >1%, as estimated by VerifyBamID^93^.
- If the number of SNVs called in a tumor was a low outlier for the assigned tumor group. Outliers were defined as tumors where the *z* score of the log number of SNVs was <−3, considering only tumors from the same tumor group.

Duplicate tumor samples were also removed to ensure that no individual was represented more than once in a tumor group. If multiple sequenced tumor samples from the same tumor group were available for an individual, we retained primary tumor samples with the highest purity, as estimated by Ccube^94^. Based on these criteria, 10,983 tumor samples were suitable for analysis—10,198 primary tumors, 634 metastases and 151 recurrences of primary tumors from 10,975 individuals. Eight individuals were represented in multiple tumor groups.

### Mutational signature extraction and deconvolution

Integer matrices of mutation counts, with a row for each sample and a column for each mutation class, are used as inputs to independent signature extractions. A total of 80 extractions are run across the five mutation types and 16 tumor tissue types.

For the classification of SBS signatures, 96 classes have conventionally been used, composed of six base substitutions (C > A, C > G, C > T, T > A, T > C and T > G) and the flanking 5′ and 3′ bases^4,7^. We extended this scheme to 288 classes by considering the transcriptional context of mutations, whether mutations fell on the transcribed, untranscribed or nontranscribed strand^10^. As per COSMIC, we classified DBS signatures into 78 classes and small ID signatures into 83 classes according to whether the variant was a deletion or an insertion, variant length, number of reference sequence repeats, and whether the variant is microhomologous. CN signatures were assigned to 48 mutation classes according to length of sequence, CN change and whether there was LOH^17^, SVs were assigned to 32 classes based on type and size of the SV and whether it was part of a cluster^9^.

Signatures were extracted de novo using a parallelized version of SPE^10^ (GitHub, version dbb9049). For all signature classes, SPE was run using random non-negative matrix factorization (NMF) initialization, Gaussian mixture model matrix normalization, 10,000 minimum and 1,000,000 maximum NMF iterations and by minimizing an objective function based on generalized Kullback–Leibler updates. SPE was applied to each cancer type separately, using between 1 and 25 SBS and ID signatures, between 1 and 20 DBS and between 1 and 15 CN and SV signatures. For SBS, DBS, ID and CN signature classes, the optimal number of signatures was chosen by considering the average stability across NMF replicates and rank-sum tests between acceptable solutions^7^. Deconvolution of SV signatures, especially in breast, ovarian and uterine cancers, recovered multiple single-class signatures that are undesirable as single classes are no more informative than the underlying mutation rates. We used the Akaike Information Criterion (AIC) to obtain an optimal solution for SV signatures, which reduced the number of single-class signatures.

The AIC is an estimate of the evidence of a model given the data. The number of parameters is $K\left(n+M\right)$, where $K$ is the number of signatures, *n* is the number of samples and *M* is the number of mutation types. The mutation rates in channels for each sample are assumed to be Poisson distributed with mean given by the expected mutation rate from the product of signatures ($S$) and activities ($A$), whereby:

$$\mathrm{AIC}=2K{(}n{+}M{)}-2\left(M\log \left({S}^{T}A\right)-{S}^{T}A\right).$$

The model with the minimum AIC has the most signatures. This downweights solutions with many single-mutation class signatures, as they did not provide much more information than the input mutation counts. The AIC for different numbers of signatures is shown in Supplementary Fig. 13 for SVs in breast, uterus and ovarian cohorts. The best AIC solution can be vastly different from solutions picked by SPE.

### Signature combiner

Signatures were extracted and decomposed to COSMIC reference signatures (for SBS, v3.3 for DBS, ID and CN, v3.2) in 16 cohorts (Supplementary Table 2). However, mutational processes can act in multiple tumor types so we combined the cohort-specific results to generate a single set of pan-cancer signatures.

First, all signatures were decomposed into the reference list where possible, with cos(sim) > 0.8. The cosine similarity between all remaining pairs of new signatures was calculated. Starting with the highest cosine similarity pair, if (1) cos(sim) > 0.8, (2) the signatures were extracted from different cohorts and (3) neither signature is already in a set, a new set was created containing the two signatures. Other new signatures were added to this set if (1) cos(sim) > 0.8 with all members of the set and (2) they were not extracted in the same cohort as any member of the set. A single signature was then generated to describe this set from the inverse-variance weighted mean of signatures in the set

$${{S}_{j}}^{\mathrm{set}}=\frac{1}{{\sum }_{j=1}^{m}{\sum }_{i=1}^{n}{w}_{{ij}}{S}_{{ij}}}\mathop{\sum }\limits_{i=1}^{n}{w}_{{ji}}{S}_{{ji}}$$

where ${w}_{{ji}}$ is the inverse variance of the $j\mathrm{th}$ mutation class of signature *i* from SPE and the signature is normalized. This process was repeated for all signature pairings with cos(sim) > 0.8, producing multiple sets of signatures.

The set with the smallest maximum cosine similarity to any reference signature was added to the reference list. This produced a new reference list with one additional signature. We then returned to the start and decomposed all signatures to the new reference list. This iterative process continued until all signatures could be decomposed into the reference list with cos(sim) > 0.8.

### Genomic alterations

Clusters of chromothripsis, chromoplexy, tandem duplications and kataegis were determined as described in the above section on calling and classifying somatic SVs. The numbers of each of these were used as input to the association model. Sample ploidy was estimated using Battenberg^67^ and used to determine whether a sample had WGD.

### DNA repair gene inactivation

Associations were tested between signature activities and repair gene-inactivating mutations. A list of DNA repair genes was taken from the overlap between tumor suppressor genes in the COSMIC Cancer Gene Census of known cancer-associated genes^95^ and the list of genes directly involved in DNA repair mechanisms from https://www.mdanderson.org/documents/Labs/Wood-Laboratory/human-dna-repair-genes.html#Human%20DNA%20Repair%20Genes (refs. ^96,97^; genes labeled as ‘defective in diseases associated with sensitivity to DNA-damaging agents’ or ‘other conserved DNA damage response genes’ were not included). This resulted in a list of 41 cancer-associated DNA repair genes.

Germline mutations for each sample in genes were taken from aggV2 in Genomics England (https://cnfl.extge.co.uk/pages/viewpage.action?pageId=156601552). Any mutations with CADD > 20 or >30 (using CADD v1.6 (ref. ^98^)) and not classified as ‘benign’ or ‘likely benign’ in ClinVar^99^ were considered as a mono-allelic inactivation of one copy of the gene. The distribution of CADD scores for ClinVar variants is shown in Supplementary Fig. 14 separated by the clinical significance annotation in ClinVar. The threshold of CADD > 20 collects the vast majority of pathogenic and only small number of benign variants; however, it also selects many missense variants of unknown significance. Therefore, the germline mutation rate is higher than is often quoted in the literature from only nonsense and frameshift mutations. The threshold of CADD > 30 also excludes most low-confidence mutations at the expense of some pathogenic variants, leading to a more conservative set of mutations.

OncoKB (v3.14)^100^ was run on somAgg VCFs in Genomics England to find the number of mutations in a gene for each sample that were classified as ‘oncogenic’ or ‘likely oncogenic’. Battenberg^6^ was used to estimate CN variants across the genome. If any region overlapping the gene in the sample had LoH in >50% of the tumor sample, this was counted as an inactivation of one allele.

LoH and germline mutations were treated as binary variables. For many genes, two germline mutations would result in the participant having a congenital condition that would make them unlikely to be a cancer patient in later life in the Genomics England cohort. Somatic mutations could take any number.

The gene inactivation parameter was the sum of germline (CADD > 20), LoH and somatic variants, to a maximum of 2. Most of the mutation burden was from germline or LoH hits as shown in Supplementary Fig. 15; however, somatic mutations are prevalent in cohorts where the gene is a known driver, for example, BRCA2 in ovary or MSH6 in CRC and uterus. In these cases, the somatic mutations are likely to be under positive selection for the given tumor type.

### Patient therapy exposure

Chemotherapy and radiotherapy can induce mutations through DNA damage^37,101^. Genomics England provides treatment information collected from PHE–NCRAS for participants as described in the clinical data section. A sample was considered to be exposed to a treatment if the treatment start date was before the tumor sampling date, which was encoded as a binary variable. The number of patients in each tumor group exposed to each type of therapy is shown in Supplementary Fig. 16.

### Associations between signature activities and therapy exposure

Signature activities of the 41 tumor types were modeled independently. Participants were included if age at sampling, sex and principle components of germline variants were available and the sample was labeled as a primary tumor or a recurrence of a primary tumor (except in the case of melanomas where the sample could be metastatic provided that the primary site was also a melanoma). The 157 participants with chronic lymphocytic leukemia were excluded. The final sample list includes 9,946 tumor samples.

Five covariates were used—log(age), sex (0 = male, 1 = female) and the first, second and third components of the principal component analysis performed on 55,603 Genomics England participants (https://cnfl.extge.co.uk/display/GERE/Principal+Components+and+genetically+inferred+relatedness). Covariates were normalized to zero mean, unit variance. As signature activities are overdispersed counts, the data were modeled using an NB generalized linear model (GLM) or, where this failed to converge, a Poisson GLM (which we will refer to as the NB/P model). We also separately used logistic regression model on the binary parameter

$${B}_{i}=\left(0\,{\mathrm{if}}\,{{\rm{A}}}_{{\rm{i}}}=0\,{\mathrm{or}}\,{{\rm{A}}}_{{\rm{i}}} < {\mathrm{median}}(\left({\rm{A}}\right)),1\,{\mathrm{otherwise}}\right)$$

where ${A}_{i}$ is the signature activity of sample *i* and the median is estimated over the samples in the tumor type.

The fitted coefficient of the NB/P model is the rate of change of the expected log activity with respect to the normalized covariate

$$\beta =\frac{\partial \log (\mu )}{\partial X}$$

where $X$ is the normalized covariate and $\mu$ is the expected mutation rate due to the signature. For the logistic model, it is the rate of change of log-odds of the signature activity being nonzero with respect to the renormalized covariate. Dividing $\beta$ by the square root of the covariance of the covariate in the group gives the rate of change of log rate with respect to the unnormalized covariate.

*P* values for the logistic regression model were evaluated using a Wilks’ likelihood-ratio test. Signature activities are not well represented by NB/P distributions, leading to inflated association significance in the NB/P model. The significance of association for the NB/P model was estimated with dCRT^73,74^. The signature activity was first modeled as a function of covariates and the target variable (for example, treatment exposure) to generate association coefficients. The target variable was also modeled as a function of all other covariates. In the case of treatment exposures, this was a binomial model with parameters fit using logistic regression

$${X{\prime} }_{\mathrm{Treatment}} \sim \mathrm{Binom}\left(\mathrm{expit}\left(Q\alpha \right),n=1\right)$$

where *Q* is the design matrix of covariates for all samples of the tumor type where $\alpha$ are the logistic regression fitted parameter values. The target variable values were then resampled from this model 100 times and the *z* score for the association with the signature activity was calculated for each resample as

$${Z}_{\mathrm{Null}}=\frac{d\log (L(X{\prime} ;\beta )}{d\beta }\times \frac{1}{I(X{\prime} ;\beta )}$$

where the likelihood, *L*, is for an NB/P and $I$ is the Fisher information and X′ are the resampled target values. The *z* score of the real target variable data is calculated in the same way

$${Z}_{\mathrm{Alt}}=\frac{\mathrm{dlog}(L(X;\beta )}{{\rm{d}}\beta }\times \frac{1}{I(X;\beta )}$$

where *X* is the target variable. The *P* value of association was calculated by comparing the alternative *z* score with the distribution of null *z* scores using a chi-square test with one degree of freedom based on the mean and variance of the null *z* scores.

### Associations between signature activities and DNA repair gene inactivation

Signatures were modeled against DNA repair gene inactivation using the NB/P model with dCRT to evaluate effect sizes and *P* values as described above. However, the gene inactivation parameter is not binary and must be modeled appropriately. Germline (CADD > 20) and LOH were resampled from an *n* = 1 binomial GLM

$$\begin{array}{l}{X{\prime} }_{\mathrm{Germline}} \sim \mathrm{Binom}\left(\mathrm{expit}\left(Q{\alpha }_{G}\right),n=1\right),\\ {X{\prime} }_{\mathrm{LoH}} \sim \mathrm{Binom}\left(\mathrm{expit}\left(Q{\alpha }_{\mathrm{LoH}}\right),n=1\right),\end{array}$$

and somatic hits from a Poisson GLM

$${X{\prime} }_{\mathrm{Somatic}} \sim \mathrm{Poisson}\left(\exp \left(Q{\alpha }_{S}\right)\right)$$

where *Q* is the design matrix of covariates for all samples of the tumor type and ${\alpha }_{i}$ are the parameters fitted to the logistic models and Poisson GLM. The resampled inactivation parameter is the sum of resampled hits with a maximum of 2

$$X{\prime} =\min \left(2,{X{\prime} }_{\mathrm{Germline}}+{X{\prime} }_{\mathrm{LoH}}+{X{\prime} }_{\mathrm{Somatic}}\right).$$

This was used to generate the null distribution of *z* scores as described in the previous section. This method reduced the false-positive rate under mock tests (see ‘Signature and gene inactivation mock tests’) within the expected distribution of *P* values as shown in Supplementary Fig. 17, where a direct estimation of significance using a Wilks’ likelihood-ratio test would produce a large false-positive rate.

We also computed associations between signature activities and only germline or somatic point mutations to produce a clearer picture of what might be driving the signature. In these cases, we used the same approach described above, modeling the germline mutation with a binomial distribution with $n=1$ and the somatic point mutations with a Poisson distribution. In this case, we do not limit the number of somatic mutations to 2 but allow it to take any non-negative integer value. For the germline associations, we used the more conservative CADD > 30 set.

### Signature and gene inactivation mock tests

Mock signatures and mock gene inactivations were generated to test for false positives. Three types of mock were generated:

1. Signature activities were simulated for all samples in the dataset from a NB model
  $${A}_{\mathrm{mock}} \sim \mathrm{NB}\left({Q}^{T}\alpha ,\theta \right)$$
  where *Q* is the design matrix of covariates, $\alpha$ are the coefficients and $\theta$ is the size parameter of the NB. Covariate coefficients were set to 1 and $\theta =1$ but there was no dependence on the gene inactivation parameter. This model has no hypermutation of samples and can be fitted well by the NB GLM.
2. Gene inactivation values were simulated in three components for the germline, somatic and LoH mutations
  $$\begin{array}{l}{X}_{\mathrm{Germline}} \sim \mathrm{Binom}\left({p}_{\mathrm{Germline}},n=1\right),{X}_{\mathrm{Somatic}} \sim \mathrm{Poisson}\\ \left({{\rm{\mu }}}_{\mathrm{Somatic}}\right),{X}_{\mathrm{LoH}} \sim \mathrm{Binom}\left({p}_{\mathrm{LoH}},n=1\right)\end{array}$$
  where ${p}_{\mathrm{Germline}},{\mu }_{\mathrm{Somatic}}$ and ${p}_{\mathrm{LoH}}$ were all set to the same value of 0.05, 0.2 and 0.5, respectively, for three separate mocks. The gene inactivation parameter was given by
  $$X=\min \left(2,{{{X}}}_{\mathrm{Germline}}+{{{X}}}_{\mathrm{LoH}}+{{{X}}}_{\mathrm{Somatic}}\right).$$
  By simulating the gene inactivations but using the true signature activities, we tested the model on a dataset that is not well represented by an NB/P GLM. However, this is akin to the resampling in dCRT, which means that the method should not produce false-positive inflation by construction.
3. Gene inactivation mocks were generated by perturbation resampling gene inactivations within each tumor type. This was performed for NTHL1, BRCA2 and MGMT, which had different mutation rates in the different tumor types. These mocks tested whether the method was robust against both hypermutated samples and any variation in the distribution of gene inactivations away from the assumed model used in the dCRT described above.

The three mocks test the method in different ways and can be used to estimate the false-positive rate in the results. Supplementary Fig. 17 shows *P–P* plots for the respective mock tests demonstrating the significant reduction in false-positive detection achieved using dCRT compared with Wilks’ likelihood-ratio test.

### Association between signatures and genomic alterations

The relationship between signature activity and genomic alterations was estimated in the same way as for treatment exposure described above. WGD was treated as a Bernoulli-distributed random variable when resampling. The numbers of chromothripsis, chromoplexy and tandem duplication events were treated as Poisson-distributed random variables when resampling.

### Associations between signatures and tumor histologies

The status and clinical features of tumors across different cohorts were retrieved from PHE/NCRAS. TNM stage was used along with NPI for breast cancers, Dukes for CRC, FIGO for ovarian and uterus tumors, Gleason for prostate and Breslow for skin melanomas. In all cases, the grade and stage were treated as continuous variables with stage given values of 0, 1, 2, 3, 4 and grade as 1, 2, 3, 4. We use continuous rather than ordinal variables, as the regression approach does not provide a method for appropriately handling them. ER, PR and HER2 hormone statuses were acquired for breast cancer participants and also binarized such that N = 0, P or Pm = 1 and any other value is treated as missing.

Associations were performed against the signature activities using logistic regression controlling for age, sex and population principal components of each participant. Missing data reduce the sample size we have to work with. Of the 9,911 samples with age, sex and principal component information that pass quality control, 7,347 had stage and 6,584 had grade recorded.

Each regression was repeated with and without the clinical feature included as a covariate and Wilks’ likelihood-ratio test generated the *P* value of association (Fig. 6).

### Timing of mutations

Mutations, split into clonal and subclonal, and further into early clonal and late clonal, were decomposed into signatures to determine subclonal and late fractions for each signature in each tumor group (Fig. 7a and Extended Data Fig. 5a). Ranking the subclonal and late fractions across samples in the tumor group produces a comparative picture of mutation subclonality relative to other signatures (Fig. 7b and Extended Data Fig. 5b).

MutationTimeR is applied to all SBS, DBS and ID mutations to classify them as clonal or subclonal. For each sample, the number of clonal and subclonal mutations is aggregated by mutation type to the 96, 78 and 83 classes used for SBS, DBS and ID, respectively. We cannot say deterministically whether any one mutation is caused by a particular signature if there are multiple signatures active in the sample that can cause the mutation type. However, by weighting mutations based on the expected fraction of the mutation type caused by each signature in that sample, it is possible to estimate the fraction of mutations corresponding to each signature^72^.

The fractional contribution of each mutation class in each sample to any given signature is evaluated from the signature distributions and activities

$${\rho }_{{ckp}}=\frac{{S}_{{ck}}{A}_{{kp}}}{{\sum }_{k=1}^{K}{S}_{{ck}}{A}_{{kp}}}$$

where *c* is the mutation class, *k* is the signature and *p* is the participant sample. These fractions are used to weight contributions from the clonal/subclonal mutation counts to estimate the number of clonal and subclonal mutations contributed by each signature

$${N}_{{kpt}}=\mathop{\sum }\limits_{c=1}^{C}{\rho }_{{ckp}}{M}_{{cpt}}$$

where *M* is the number of mutations and *t* is 0 for clonal or 1 for subclonal. The fraction of subclonal mutations corresponding to each signature in each sample is ${f}_{{kp}}={N}_{{kp}1}/\left({N}_{{kp}0}+{N}_{{kp}1}\right)$. Mann–Whitney *U* tests were then used to compare $f$ for the given signature compared to all other signatures in the tumor group. The mean rank of the signature is estimated by ranking all $f$ in the tumor group and taking the mean of ranks of the given signatures. If this is less than 0.5, the signature is relatively early in the given group, if the mean rank is greater than 0.5, then the signature mutations occur relatively late.

### Survival analysis

Survival time for each participant was measured from the date of tumor sampling to the date of most recent follow-up or death. We used a CPH model for each tumor type implemented in the Python lifelines package with age, sex and principle components as covariates and included tumor grade encoded as a numerical value from 1 to 4. TNM stage was not included as a covariate as this failed proportionality assumptions across multiple cohorts. Signature activities were transformed to $\log (\mathrm{activity}+1)$ and were also used as covariates when being tested. All variables were normalized to zero mean, unit variance before fitting the model.

In all cases, we ran proportional hazard and Ljung–Box tests, and we report results only when the proportional hazard test of the signature coefficient has *P* > 0.01.

### Aggregated signatures

To produce Extended Data Fig. 6, associations were aggregated across tumor types. For the gene inactivation, genomic alteration, treatment and clinical associations both results from dCRT on signature activities in samples and logistic regression on nonzero signature activity were used. For survival, Wilks’ *P* values were estimated using the CPH likelihood and for mutation timing the Mann–Whitney *U* test *P* values were used. Only results with a study-wide significant (Benjamini–Hochberg FDR = 0.01) association in at least one association test were considered. Each signature and target variable was also only included if the significant associations were all either positive or negative across cohorts. In cases of DNA repair gene inactivation, only positive associations were considered to reduce confounding. For cases where multiple tests were considered, the combined *P* value was estimated by taking the binomial probability that at least one of multiple tests would receive a *P* value less than the minimum of the set. *Z* scores were calculated by taking the square root of the inverse-survival function for a chi-square test with one degree of freedom of the *P* value

$${Z}_{i}^{2}=\mathrm{CD}{{\rm{F}}}_{{\chi }^{2}}^{-1}\left(1-{P}_{i}\right).$$

These were summed across tumor groups to obtain the total residual sum of squares, which was used as a chi-square statistic with the same number of degrees of freedom as groups. Due to confounding between different targets, for example, correlations between inactivations of different genes, we discard any result with an overall *P* value that is not within a factor of 20 of the minimum *P* value for the given signature within that set of associations. This is then converted back to an aggregated *z* score as in the method above, with a single degree of freedom.

The *z* scores evaluated this way are shown in Extended Data Fig. 6 of the present study.

### Reporting summary

Further information on research design is available in the Nature Portfolio Reporting Summary linked to this article.

## Online content

Any methods, additional references, Nature Portfolio reporting summaries, source data, extended data, supplementary information, acknowledgements, peer review information; details of author contributions and competing interests; and statements of data and code availability are available at 10.1038/s41588-025-02474-x.

## Supplementary information

Supplementary Information Supplementary Notes 1–6 and Supplementary Figs. 1–17.

Reporting Summary

Supplementary Tables 1–13. Summary statistics of Genomics England cohort data and signature analysis.

## Data Availability

Data from the NGRL used in this research are available within the secure Genomics England Research Environment. Access to NGRL data is restricted to adhere to consent requirements and protect participant privacy. Data used in this research include: BAM files and VCF files containing SNV, indel and SV calls. The corresponding metadata and file locations for these files can be obtained through LabKey by querying the ‘cancer_analysis’ table. Processed clinical and genomic data, available in the Research Environment within the folder /re_gecip/shared_allGeCIPs/pancancer_signatures. To support reproducibility, a README file listing all participants’ IDs used is included in /re_gecip/shared_allGeCIPs/pancancer_signatures. At present, there is no proposed end date for data access within the research environment. All other public/private datasets used in the study, including corresponding download links and version numbers, can be found in Supplementary Tables 1–13. Access to NGRL data is provided to approved researchers who are members of the Genomics England Research Network, subject to institutional access agreements and research project approval under participant-led governance. The raw data, including patient profiles and corresponding genomic sequencing data, are available under restricted access for patient privacy reasons. Access can be obtained by first applying to become a member of either the Genomics England Research Network (https://www.genomicsengland.co.uk/research). The process for joining the network is described at https://www.genomicsengland.co.uk/join-us and consists of the following steps: Your institution will need to sign a participation agreement available at https://files.genomicsengland.co.uk/documents/Genomics-England-GeCIP-Participation-Agreement-v2.0.pdf and email the signed version to gecip-help@genomicsengland.co.uk. Once you have confirmed your institution is registered and have found a domain of interest, you can apply through the online form at https://www.genomicsengland.co.uk/join-us, where you can specify the reason for access and expected time frame that you wish to have access. Once your Research Portal account is created, you will be able to log in and track your application. Your application will be reviewed within 10 working days. Your institution will validate your affiliation. You will need to complete the online Information Governance training and will be granted access to the Research Environment within 2 days of passing the online training. The Research Environment is accessed through Amazon WorkSpaces (https://clients.amazonworkspaces.com/). For more information on data access, visit https://www.genomicsengland.co.uk/research.

## Code availability

Supplementary Table 13 lists software used in this work and SPE parameters used for signature extraction. Code used for analysis is provided in the Genomics England Research Environment under /re_gecip/shared_allGeCIPs/pancancer_signatures/code. This code has also been made publicly available in a dedicated GitHub repository at https://github.com/Wedge-lab/Gel_pan_cancer_signatures (10.5281/zenodo.17709379).
