# Supplementary material for: Comprehensive repertoire of the chromosomal alteration and mutational signatures across 16 cancer types
Source: Nat Genet. 2026 Feb 13;58(3):570–81. doi: 10.1038/s41588-025-02474-x (PMC12987726; doi:10.1038/s41588-025-02474-x)
Supplement: Supplementary file 1 — Supplementary Notes 1–6 and Supplementary Figs. 1–17. [file 41588_2025_2474_MOESM1_ESM.pdf]

nature genetics

Supplementary information https://doi.org/10.1038/s41588-025-02474-x

Comprehensive repertoire of the chromosomal alteration and mutational signatures across 16 cancer types

In the format provided by the authors and unedited

Supplementary information

Supplementary Note 1. Single and Doublet Base Substitution Signatures 1
Supplementary Note 2. Insertion and Deletion (InDel) signatures 2
Supplementary Note 3. Mechanistic basis of signatures 2
Supplementary Note 4. Confounding of gene-inactivation/treatment associations 3
Supplementary Note 5. Comparison with previously extracted signatures 3
Supplementary Note 6. Clinical applicability 4
Supplementary Figures 6
REFERENCES 17

Supplementary Note 1. Single and Doublet Base Substitution Signatures

We found SBS57 clustered with signatures for dMMR (**Fig. 3**). The signature is associated with TTT>TCT single base substitutions. This is caused by insertions and deletions (InDels) occurring in long homopolymer chains, which can "trick" the variant caller into calling single base substitutions at the end of the homopolymers rather than InDels that occur somewhere in its length.

Of the 67 SBS signatures extracted as part of this work, two (SBS24 and SBS29) have no reported activity in any samples. SBS24 is extracted only in the lung cohort and SBS29 only in ovary. In each cohort we extract signatures initially without any reference comparison retrieving 16 lung and 17 ovary cancer signatures. These signatures are then decomposed into COSMIC reference signatures (where this can be done with greater than 0.8 cosine similarity, otherwise the signature is considered novel) after which activities are determined by decomposing the mutation activities into the reference (Supplementary Methods Section 8). The 3rd most active signature in the lung cohort was decomposed SBS4 with 60.4% contribution, SBS5 (24.8%) and SBS24 (14.9%). This is the only signature in lung cancers which was decomposed into SBS24. However, when decomposing mutational activities in the cohort, no activity was assigned to SBS24, likely due to the slight change in signature distributions from the decomposition. Similarly, in the ovary cohort, the 9th most active signature was decomposed into SBS1 (5.3%), SBS5 (65.4%), SBS23 (13.9%), and SBS29 (15.3%) and SBS29 was not used in the decomposition of any other signatures. After this, no activity was assigned to SBS29. In summary, some signatures were extracted due to low expected contributions to extracted signatures however these signatures were not required to produce the mutation activities in any samples.

By investigating read alignments we also found that DBS14 has many T>C and T>G mutations in repetitive T regions of the genome where the C is often displaced by a single base due to read misalignment, resulting in clonal T>C/G mutations being called as heterozygous subclonal TT>CC/GG mutations (also reported by Degasperi *et al.*[1] as DBS28). Since read-level information is not included in VCF files, such pairs of single nucleotide variants can mistakenly be identified as DBS events. An example of an artefactual DBS event is shown in **Supplementary Figure 1**. To quantify the number of such artefacts we verified DBS events against the read alignments available in the corresponding BAM files. All reads mapped to the region of the reported DBS mutation were evaluated to confirm the presence of the consecutive nucleotide variants or their reverse complement. A DBS mutation was considered genuine if at least one read contained the pair of nucleotide mutations in consecutive positions; otherwise, it was classified as an artefact mutation.

SigProfilerExtractor identified 2,151,714 DBS mutations in 10,983 samples, of which 2,004,154 (93.1%) were verified as real and 147,560 (6.9%) identified as artefacts affecting 2,177 samples (**Supplementary Figure 2**).

The DBS mutational context probabilities, as reported by SigProfilerExtractor, were used to estimate the number of real and artefact DBS mutations included in each of the extracted DBS signatures. Signatures DBS3, DBS10, DBS12, DBS14, and DBS15 were found to contain relatively large proportions of artefact mutations (>10% of the signature-related activities were identified as artefact DBS mutations) (**Supplementary Figure 2B**).

Supplementary Note 2. Insertion and Deletion (InDel) signatures

When we initially extracted InDel (ID) signatures, we used all mutations that passed quality control filters applied by Genomics England. This produced a mutation count matrix of 142,764,499 InDels across all samples. Amongst these mutations was a significant excess of single T/A deletions from TT/AA dinucleotide pairs across all samples (**Supplementary Figure 3**). This mutation type is a key feature of ID13, a UV signature, which has previously been extracted only in melanoma.

The cause of this excess of mutations are germline single base deletions in long homopolymers, which occur adjacent to a TT/AA dinucleotide pair. In the tumour, a single base substitution can occur on one of the bases in the dinucleotide pair. For any single read the exact mutation is degenerate with the scenario where a deletion happens in the dinucleotide pair with a single base substitution at the start of the long homopolymer. The Strelka variant caller typically calls the latter event as the somatic mutation that has taken place, as context from germline reads is not considered. Therefore, a germline single base deletion in a long homopolymer is incorrectly called a somatic deletion to a homopolymer pair. An example of this in a sarcoma sample is shown in **Supplementary Figure 4**.

We therefore applied an additional filter within Genomics England to remove somatic InDels within 10bp of a germline InDel present in >1% of samples in Genomics England or GNOMAD. This reduced the rate of ID13 in non-melanoma samples by ~99%. By inspecting the reads we found that remaining ID13-attributed deletions were not caused by artefacts in alignment or variant calling.

Supplementary Note 3. Mechanistic basis of signatures

In the main text we highlight some key associations found between signatures and DNA repair gene inactivation’s or treatment exposures in specific tumour groups.

We also analysed the signature activity between germline and somatically mutated *BRCA1* and *BRCA2* samples. However, classifications of mutations as oncogenic or non oncogenic can be significantly different between germline and somatic mutations. For example, when considering samples with germline mutations of CADD>20 of BRCA1 in Breast-DuctalCA, the average SBS3 activity across 270 samples was 710 compared with 3194 for those with an oncogenic (or likely oncogenic) somatic mutation as classified by OncoKB (Two sample two-sided t-test P=8.45e-6). If we use a more conservative CADD>30 constraint on the germline mutation, the mean activity for SBS3 increases to 2709 (P=0.785). Therefore, we caution against making direct comparisons between the rates of mutations caused by germline and somatically inactivated genes as there is no consistent definition for an oncogenic mutation across both scenarios.

Supplementary Note 4. Confounding of gene-inactivation/treatment associations

Along with the relationships described for gene inactivation's and treatment exposures, each signature is typically associated with multiple seemingly unrelated genes in a tumour type although usually with smaller effect sizes. This may be caused by confounding between inactivation of different genes that are highly correlated (**Supplementary Figure 5**) or reverse causation as tumours with higher mutation rates are more likely to have mutations in genes. Many chemotherapeutic agents are administered in combination such that separate treatments are also not independent (**Supplementary Figure 6**). To unambiguously ascribe signature causality requires controlled laboratory experiments or analysis of tumour recurrence[2,3].

Supplementary Note 5. Comparison with previously extracted signatures

Degasperi *et al.*[1](Degasperi22) have also extracted SBS and DBS signatures from the 100kGP data. In response to reviewers we discuss below differences between Degasperi22 and our extractions, which are a consequence of the following: *(i)* Differences in sample quality control between the two studies meant we analysed fewer samples than Degasperi22; *(ii)* For the DBS signatures, the number of DBS differs between samples*; (iii)* Model assumptions and complexity; *(iv)* Differences in the reconstructed mutation rates across samples between studies. We provide a detailed discussion below on how these differences impact the study's findings.

(i) Sample quality control

In our study, sample quality control excluded PCR amplified samples, and only those with consistent reporting of tumour groups, sample age, and sex across the Genomics England data, the 13 GMCs, PHE/NCRAS, and NHSD were analysed. Of the 10,983 samples analysed in our study, 9,328 are also included in the 12,222 samples considered by Degasperi22. Of the 2,894 samples we did not analyse, 11 are not in Genomics England version 11 (V11) (Degasperi22 used V8), 802 were PCR amplified, 19 had inconsistent sex reported, 1,326 could not clearly be assigned to a specific tumour group, and for 75 tumour type was ambiguous. Additionally, 3 had no reported date of sampling, 67 did not have age information, 47 did not pass sequencing quality control, and 578 were excluded because they were duplicate samples.

(ii) Doublet-base substitutions (DBS) counts

While SBS mutation rates are highly consistent between the two studies (<0.1% variation on average between the same samples and mutation types; **Supplementary Figure 7**), there is larger variation between the DBS mutation rates recovered. Degasperi22 recorded ~12% more DBS mutations than we identified using SigProfilerMatrixGenerator with the 'seqInfo' being set to 'True', which prevents classifying multi-base substitutions (*i.e.*, having greater 2 adjacent single base substitutions) as DBS events. This approach ensures that only doublet-base substitutions, and not multi-base substitutions, are considered when examining DBS signatures in the current study.

(iii) Model complexity

Degasperi22 decompose their data into a larger number of signatures: 85 SBS and 25 DBS signatures for the 9,328 100kGP samples considered by both studies. This compares to the 65 SBS and 19 DBS signatures extracted in our work from 10,983 samples. We have based our signature extraction process on an automated strategy with strict selection criteria for a signature to be declared novel, aiming to make our methodology generalisable to other datasets with minimal manual decision making. NMF

was applied to each tumour group and our novel signatures are included such that they have minimum similarity to those in the COSMIC reference catalogue. Degasperi22 included additional 'rare signatures' by curating outlier samples and separately extracting signature profiles. Using SigProfilerExtractor we examined whether the signatures extracted by Degasperi22 could be explained and decomposed using the COSMIC reference signatures and the novel signatures herein. Of the 40 novel SBS signatures from Degasperi22, 3 match the novel SBS signatures we identify and a further 32 can be decomposed to COSMIC signatures (i.e cos(sim) > 0.80) (**Supplementary Figure 8**). Degasperi22 reported 39 DBS signatures, 30 of which are not in COSMIC. All but one (DBS18) of the remaining signatures can be decomposed to the COSMIC reference or the 8 signatures extracted in this work (**Supplementary Figure 9**). As such, many of the signatures reported in Degasperi22 are linear combinations of previously known COSMIC reference signatures.

(iv) Reconstruction accuracy

We compared the accuracy of reconstructed sample mutation rates between our study and Degasperi22 with the caveat that, for DBS signatures, it is challenging to make a robust comparison given the differences in counting doublet-base substitutions (see above).

First, we examined the cosine similarity between the original mutation rates and reconstructed signatures (**Supplementary Figure 10**). Of the 9,328 samples used in both studies, 4,721 have better SBS reconstructions in our study than Degasperi22. Of the 7,035 samples with DBS mutations recovered by both studies, 5,011 have greater cosine similarity in our study. A second relevant metric is the KL divergence, which both studies use to measure goodness of fit between the measured data and the model (**Supplementary Figure 11**); equivalent to the negative log likelihood under Poisson distributed uncertainties. The median KL divergence in our study was 1.38 for SBS and 0.19 for DBS compared with 1.02 and 0.19 for Degasperi22, respectively, suggesting a better performance in Degasperi22 for SBS and comparable performance of the 2 methods for DBS. However, the comparison between DBS signatures is also made challenging by the fact that the variant QC applied in each study is significantly different in this mutation type leading to different mutation rates.

Our SV1-SV6 are very similar (cos(sim) > 0.90) to those reported by Nik-Zainal et al[4] in WGS analysis of 560 breast cancers. SV7, SV8 and SV9 match the signatures R7, R8 and R6b reported by Desgasperi *et al.*, 2020 in a WGS study of 3,107 cancers[5] (**Supplementary Figure 12**).

Supplementary Note 6. Clinical applicability

If signature analysis is to become part of routine patient care as envisaged by the 100kGP it is essential that analyses should be cognisant of the statistical issues surrounding assignment of signatures and potential errors both from sequencing artefacts and downstream analyses.

For signatures to be used in clinical treatment decisions, assignments must be stable and reproducible. Standardised WGS and variant calling methods are required as signature extraction is performed on mutation counts, which are a function of sequencing coverage and calling sensitivity. For example, different rates of signature activities were observed in this study compared with PCAWG, *e.g.* SBS17b is present in 22% of CRC samples in this study compared with 2% of samples reported in PCAWG[6] likely due to the greater coverage of WGS compared with the predominantly whole exome sequenced samples used in that study.

The approach taken to sample collection for each tumour group should also include consistent and robust inclusion criteria and important confounders such as tumour stage, grade, site and

histology should be reported for all samples. The 100kGP is a hugely valuable resource providing a broad picture across many cancer types and high depth sequencing. However, the vague inclusion criteria and inconsistent reporting of confounders makes it challenging to establish clinically applicable results. To complete our study, we had to unfortunately exclude 15.9% of samples due to conflicting or missing data and only a small handful of samples have recorded data on lifestyle risk factors such as smoking and BMI.

Figures

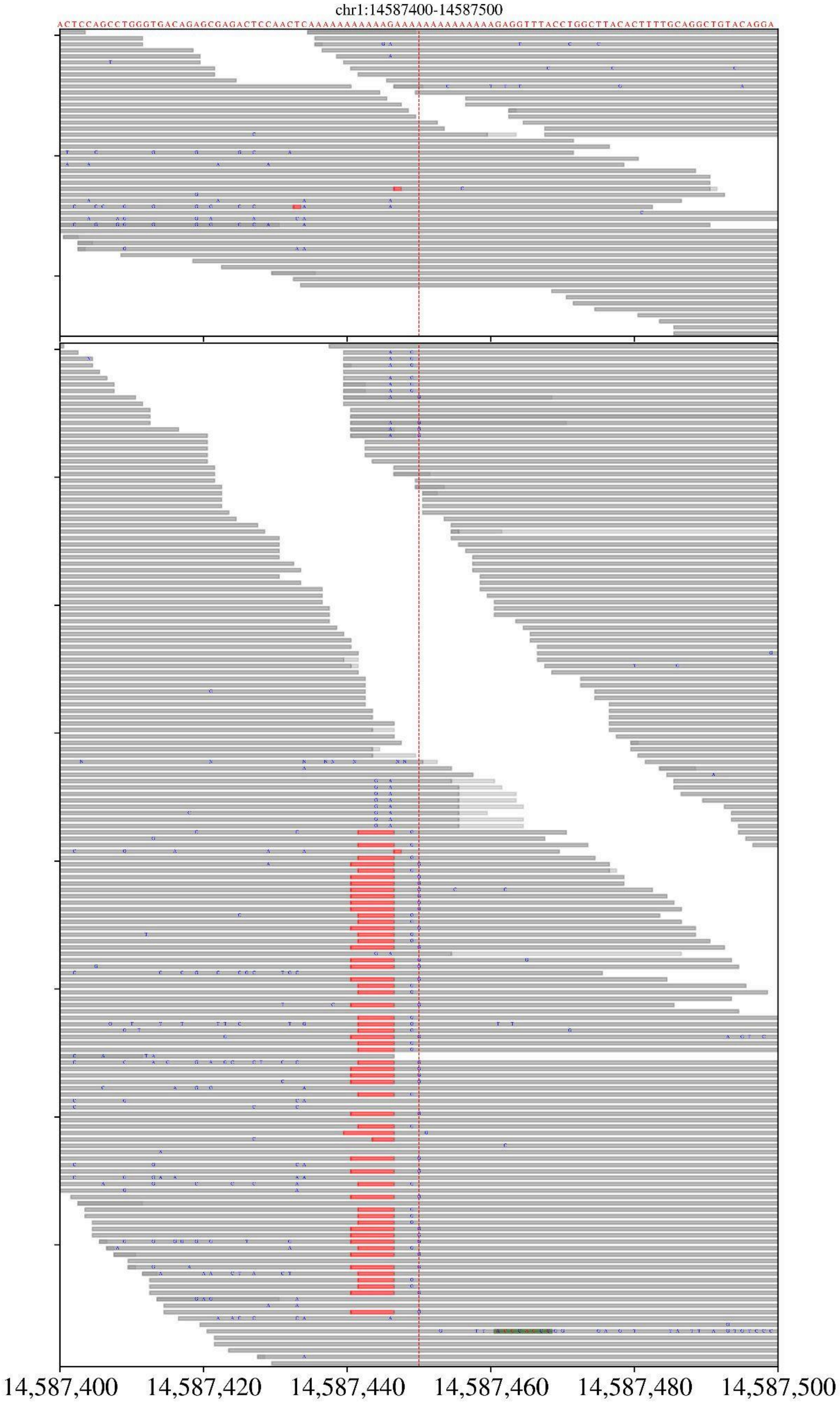

Supplementary Figure 1. A sample with a high DBS14 burden around the location at which a TT>CC mutation is called. A somatic deletion is called in approximately half of the reads; however the caller incorrectly places the deletion in the wrong position, deleting the G base in the homopolymer stretch and calling an A>G mutation in a separate position. Due to the variable length of the deletion, the A>G mutation is misaligned between reads resulting in a large fraction of reads with A>G in neighbouring positions. The pair of apparently adjacent SNVs are then called as a

dinucleotide variant AA>GG. By phasing reads, we can determine that these substitutions occur on mutually exclusive reads and filter them out.

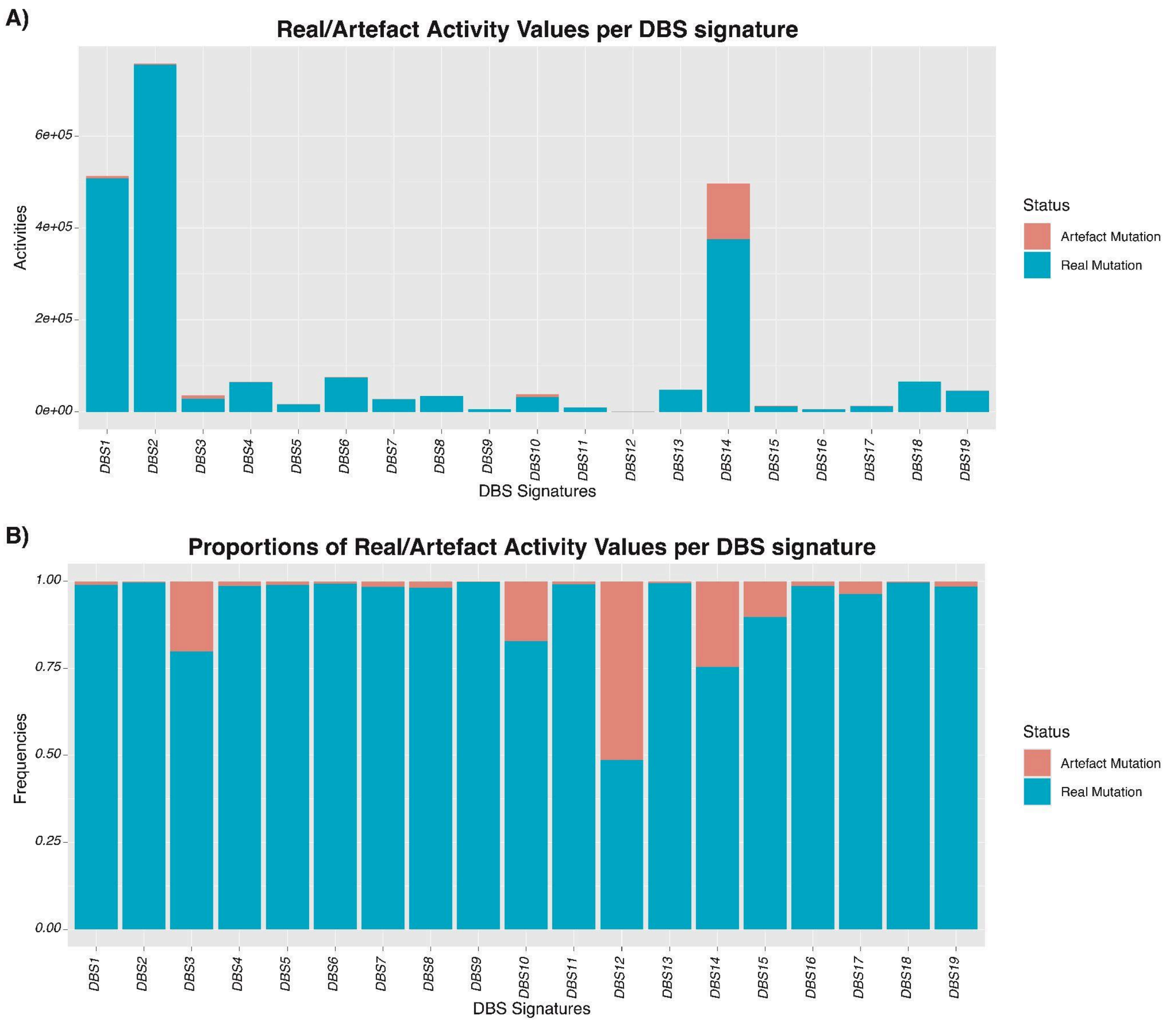

Supplementary Figure 2. Artefact DBS mutations identified across the 19 extracted DBS signatures. A) The number of real/artefact DBS mutation activities included in each DBS signature. B) Proportions of real/artefact DBS mutation activities included in each DBS signature.

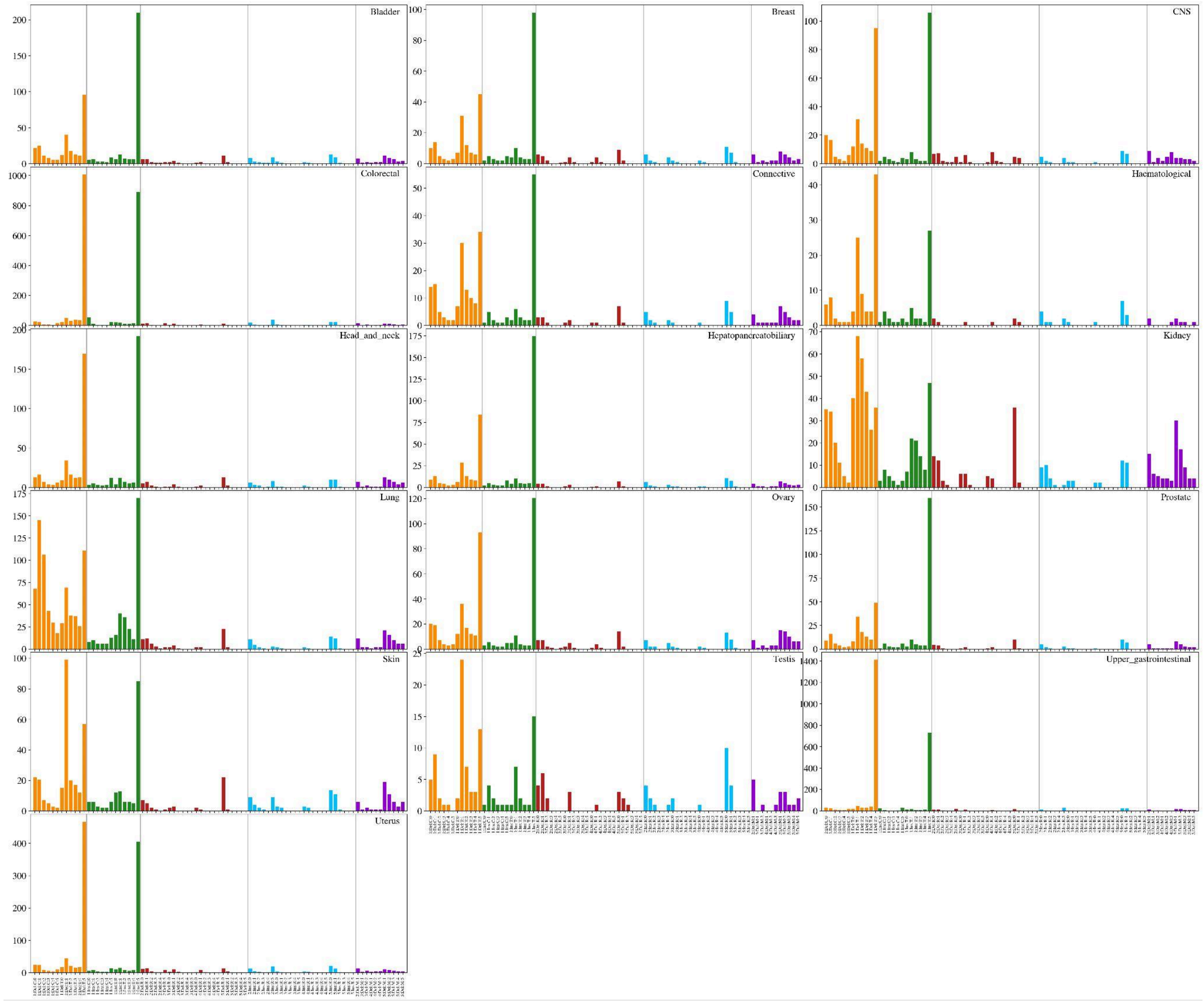

Supplementary Figure 3. The median mutation count of each ID mutation type in tumour cohorts used for signature extraction. T deletions in length 2 homopolymers are the largest contribution to skin but are also significant contributors to most other cohorts.

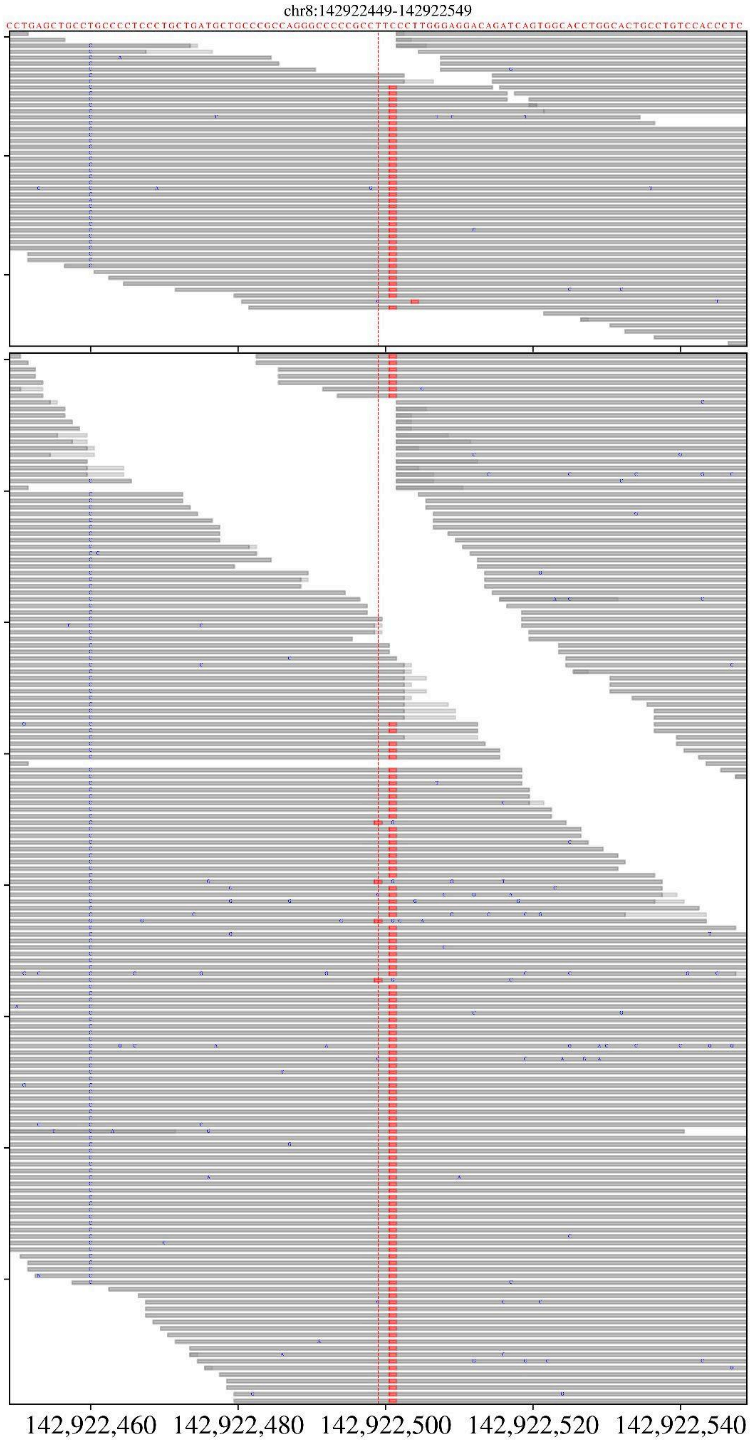

Supplementary Figure 4. An example of a mutation contributing to artefactual InDel mutations. In the top panel, the germline reads include a biallelic single base C deletion from a repeating C sequence preceded by a TT. This is called correctly for most of the tumour reads in the bottom panel, however, in four reads the single base deletion is placed in the TT while a C>G substitution is placed on the germline deletion position. For these reads, the sequence will appear as TTCCC > TGCC. This is treated as a T deletion with C>G substitution but given the germline deletion, the correct mutation would have been a T>G with a C deletion. As such the T deletion is incorrectly called as a somatic mutation. We have filtered these from the analysis by removing any InDels within 10bp of a germline InDel.

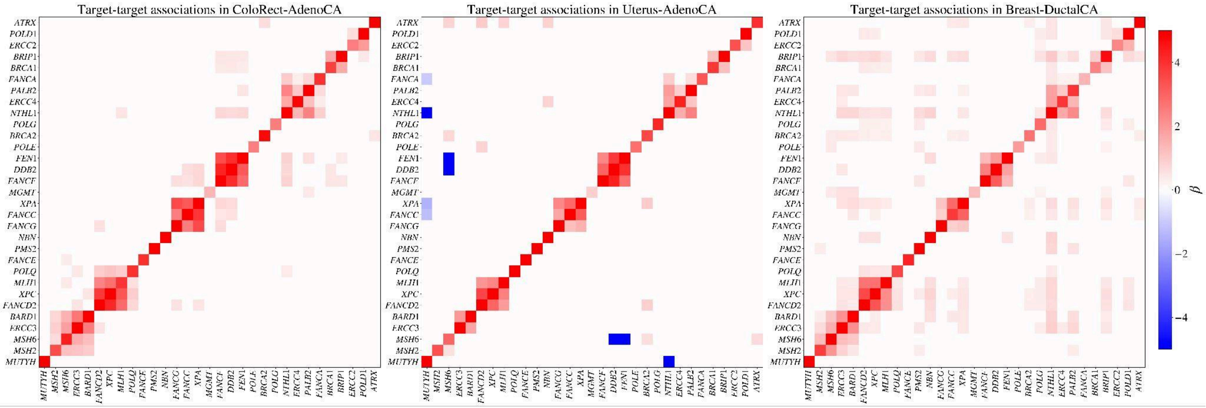

Supplementary Figure 5. The knockout of individual genes are not independent but highly correlated in many cases which can complicate interpretation of the results. The genes are associated against one another with a binomial regression with n=2 and the same 5 covariates as are used in the signature regression. The panels show the association coefficients where the chi-squared *P*-value<0.01 in three groups: CRC, Endo and DuctalCA. The genes are ordered by chromosome position and nearby genes are often highly associated forming blocks along the diagonal.

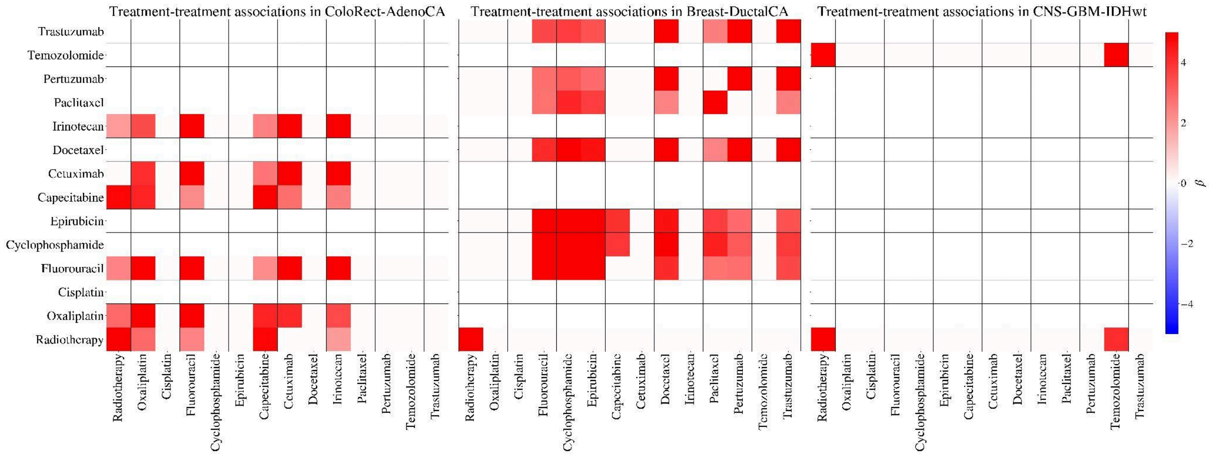

Supplementary Figure 6. As in **Supplementary Figure 5**, the associations between treatment exposures are modelled with a logistic regression to find correlations between given treatments in each cohort. CRC, DuctalCA and GBM-IDHwt are shown in the three panels of this figure. Some treatments are typically given together such as epirubicin, cyclophosphamide and fluorouracil in DuctalCA which is given as the combined treatment FEC.

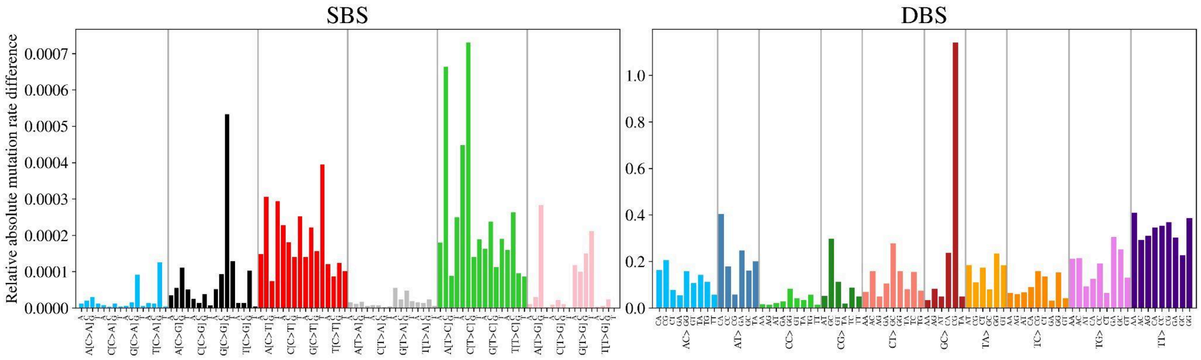

Supplementary Figure 7. The relative difference in mutation rates between this work and Degasperi22. This is evaluated as the difference in number of mutations between the two studies divided by the number of mutations found in this work. The two studies have very similar SBS mutation rates however the DBS mutation rate is much higher in Degasperi22.

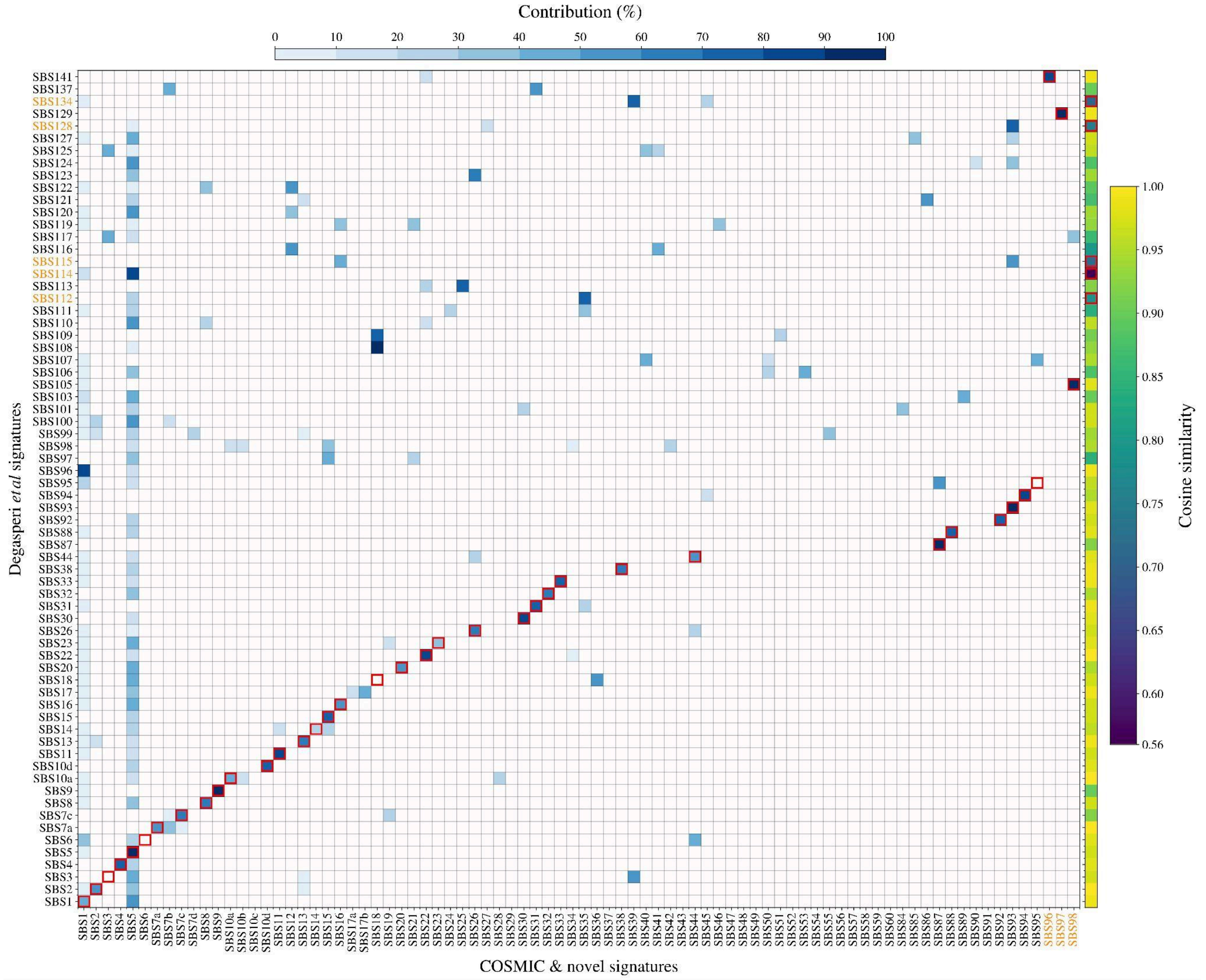

Supplementary Figure 8. Degasperi *et al*[1] SBS 'green' rated signatures (high confidence subset) are decomposed to the COSMIC v3 reference signatures and the novel signatures. The blue shading represents the % contribution of each reference signature to the Degasperi signature. The right panel shows that only 5 signatures (highlighted red) could not be decomposed with $\cos(\text{sim}) > 0.80$ between the original signature and the reconstruction. The rest wouldn't be extracted by SPE as they can be composed of linear combinations of reference signatures.

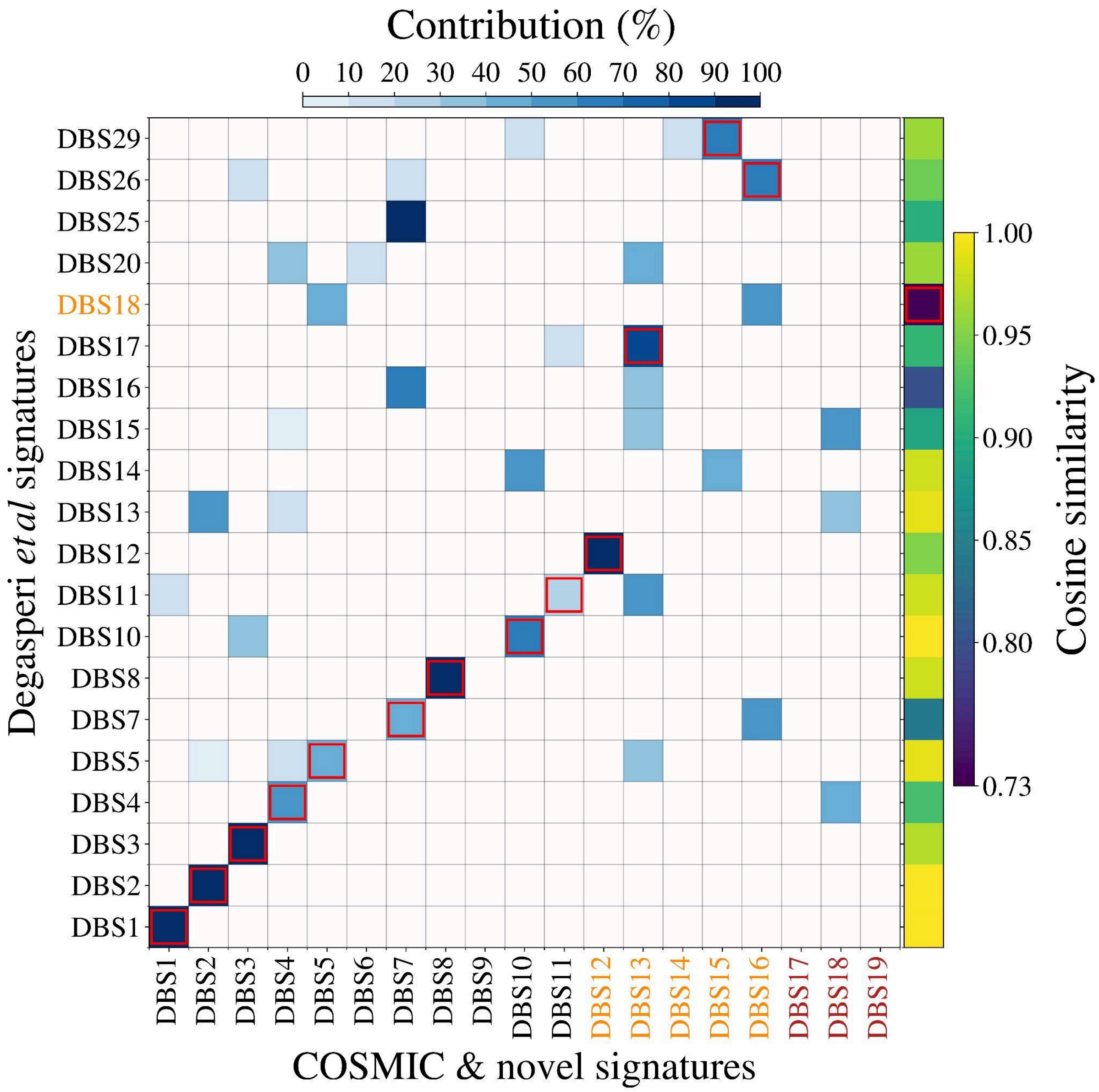

Supplementary Figure 9. As **Supplementary Figure 8** but for DBS signatures. Only one Degasperi DBS signature, DBS18, cannot be decomposed to the signatures from COSMIC and this work.

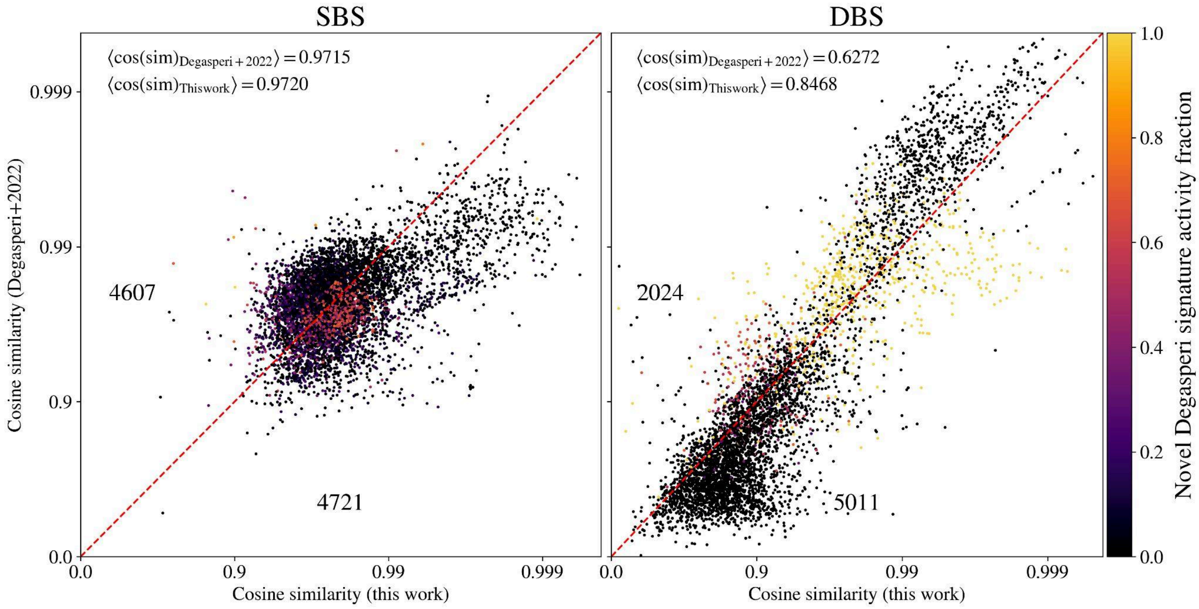

Supplementary Figure 10. The cosine similarity between SBS (left) and DBS (right) mutation

spectra of the sample and reconstructed spectra from signatures for this work (x-axis) and Degasperi22 (y-axis).

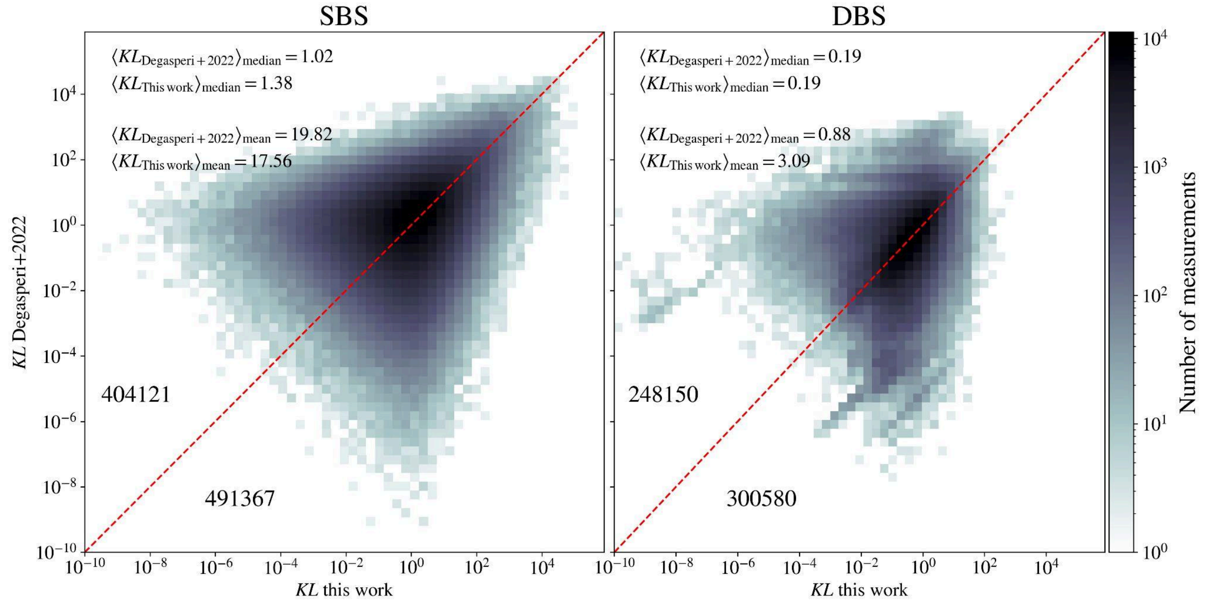

Supplementary Figure 11. The KL divergence between measured mutation counts and expected mutation counts from signature reconstructions for SBS (left) and DBS (right) mutation types in this work (x-axis) vs Degasperi22 (y-axis).

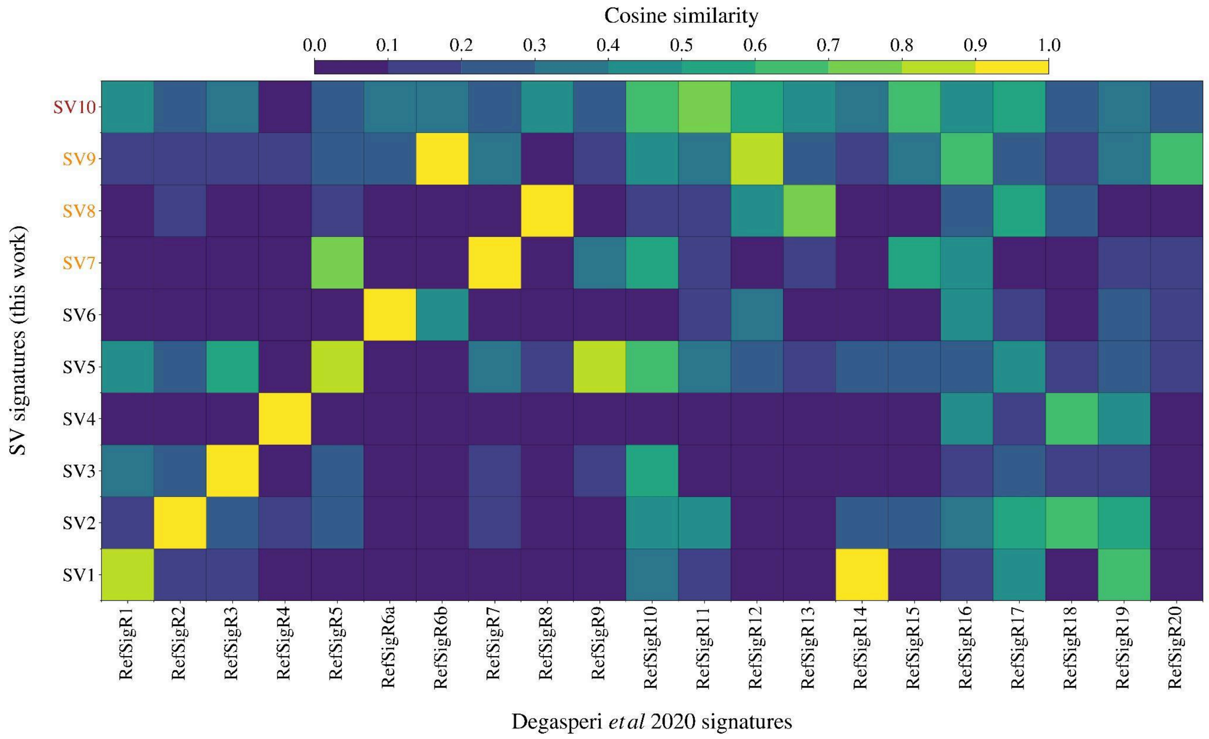

Supplementary Figure 12. The 10 extracted SV signatures are matched to previously discovered SV signatures from Degasperi *et al* 2020 based on their cosine similarity. The pairing with the highest similarity is matched where neither signature has already received another match. The red squares show the pairings which are selected where the underlying colour map is the cosine similarity of the pairs.

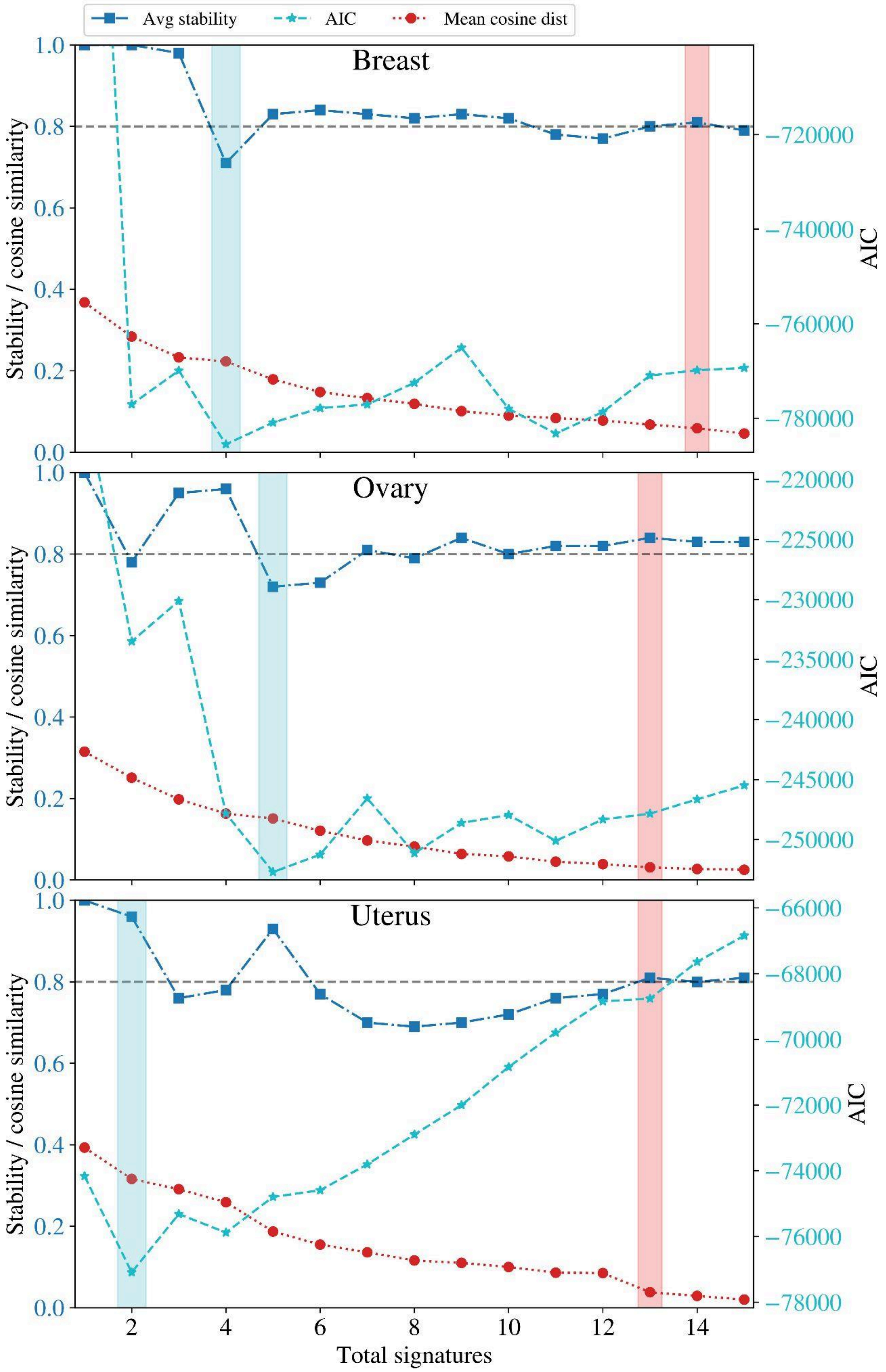

Supplementary Figure 13. SigProfilerExtractor picks large numbers of signatures as the optimal solutions for SVs in Breast, Ovarian and Uterus cohorts which include many single-channel signatures. The solution which SigProfilerExtractor picks (red shaded) has average solution stability (blue dot-dashed) greater than 0.8 and looks for significant improvements in signature concordance

related to the mean cosine distance (red dotted line). This work uses the AIC (cyan dashed) to select the optimal number of signatures (cyan shaded) in all cohorts. For the three cohorts shown, the AIC solution involves far fewer signatures than the SigProfilerExtractor recommended solution.

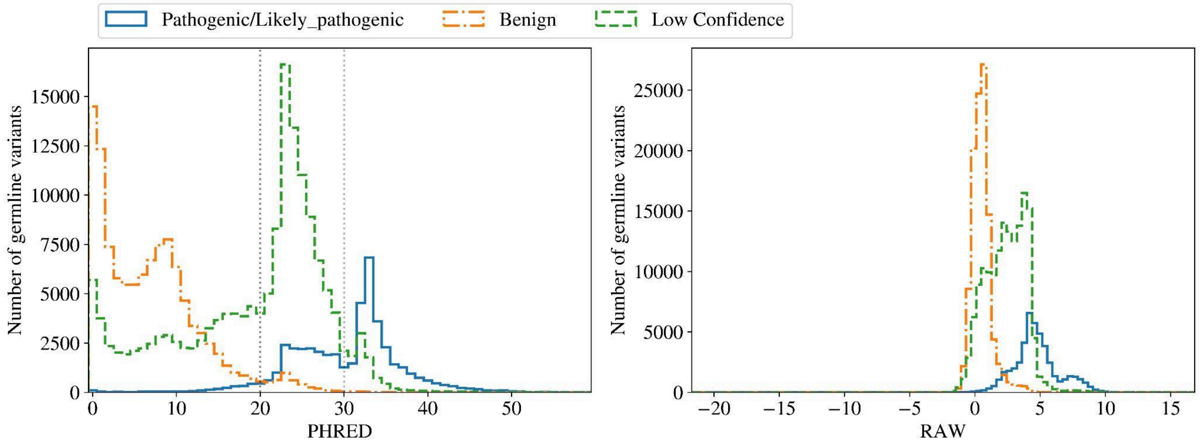

Supplementary Figure 14. CADD is run on the set of variants classified in ClinVar producing a set of RAW and PHRED scores. The histograms of these are shown above split by whether ClinVar considers the variant pathogenic, benign or if there is low confidence. A PHRED threshold of 20 removes most benign variants while retaining most pathogenic variants, which are typically nonsense or frame-shift changes. It also keeps many low confidence variants which are mostly missense mutations.

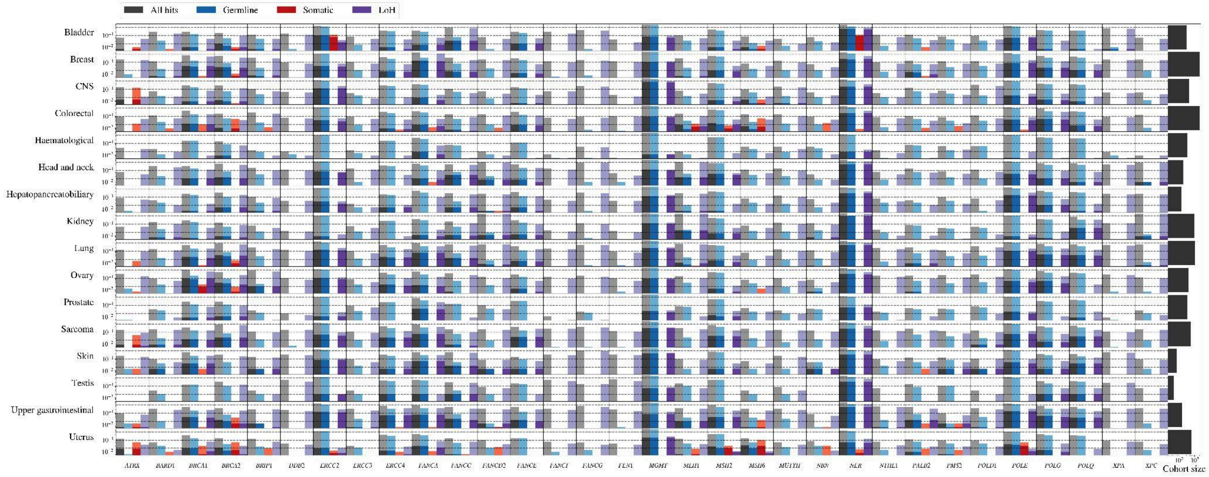

Supplementary Figure 15. Gene knockout is determined from a combination of germline, somatic and LoH type mutations. This figure shows the number of samples in each cohort which occur to each gene. The light shaded bars show the number of samples with a single hit in the germline (blue), somatic (red) and LoH (purple). The darker shading for each bar is the subset of samples with one hit of that type which also received a second hit (which may have been of a different type). The grey bars are the totals of single or double hit samples and the histograms on the right show the number of samples in each cohort.

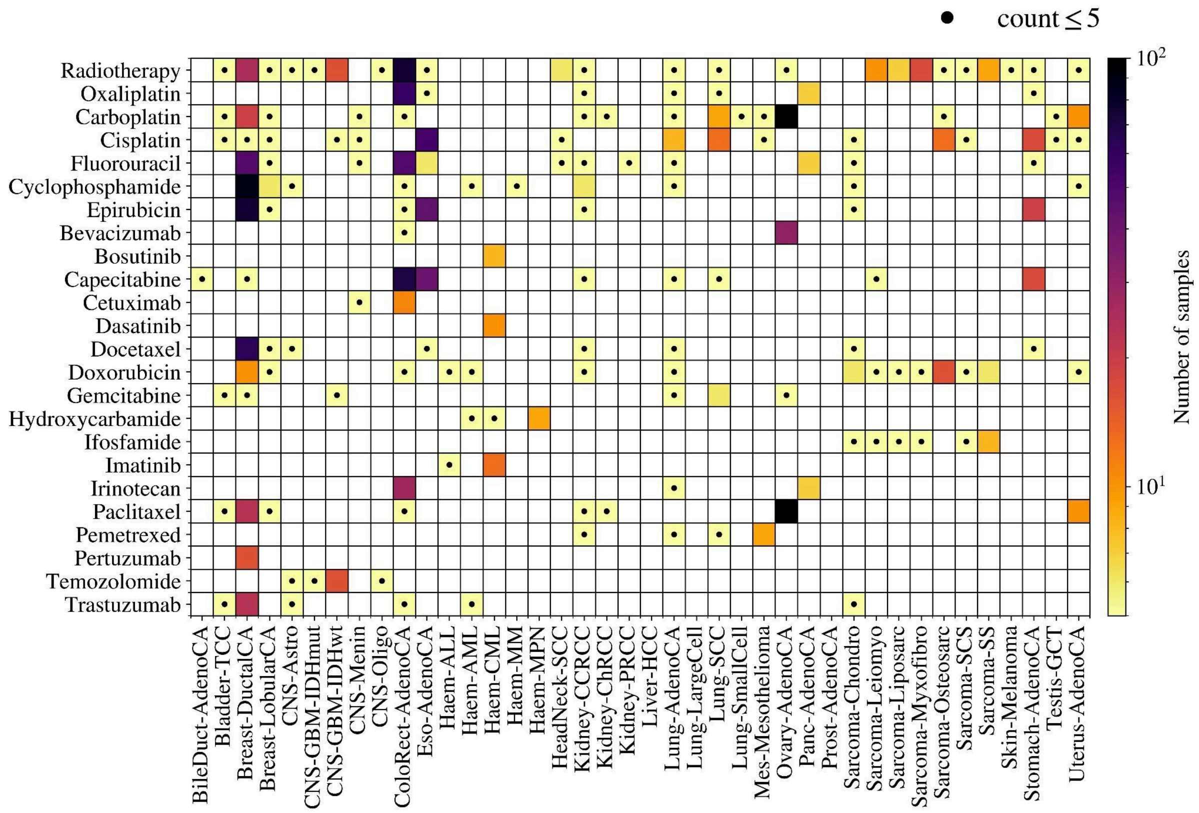

Supplementary Figure 16. Information on the treatments used for each tumour are taken from NCRAS and a sample is labelled as exposed to the treatment if the start of the course is before the sample was taken. This figure shows the number of samples exposed to each treatment type in each tumour group where combinations with 5 or fewer samples are labelled with a dot.

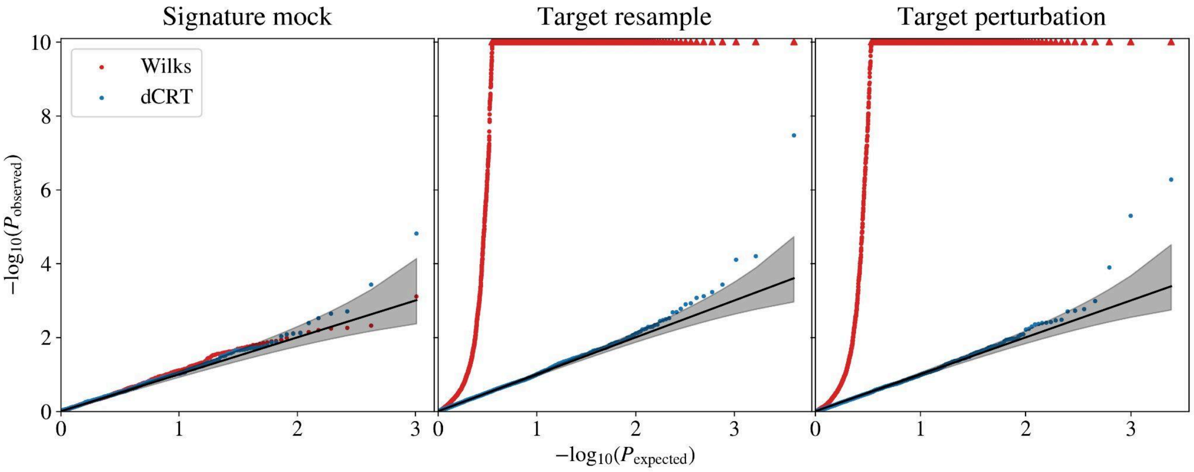

Supplementary Figure 17. Mock tests are run for the gene knockout associations using three methods: the signature is resamples from a negative binomial model, the target is resampled from the input target model and the targets undergo a perturbation resampling to reorder (**Methods**). In all cases there should be no association between the targets and signatures. The figures show Q-Q plots of expected and observed *P*-values. When applying a Wilks likelihood ratio test on the negative binomial model outputs (as is done in [7]), there is massive *P*-value inflation resulting in a large Type-I (false positive) error rate. However, conditional resampling successfully reduces this error rate to within the expected uncertainty on the distribution of p-values.

REFERENCES

1. Degasperi, A. *et al.* Substitution mutational signatures in whole-genome-sequenced cancers in the UK population. *Science* **376**, (2022).

2. Kucab, J. E. *et al.* A Compendium of Mutational Signatures of Environmental Agents. *Cell* **177**, 821–836.e16 (2019).

3. Zou, X. *et al.* A systematic CRISPR screen defines mutational mechanisms underpinning signatures caused by replication errors and endogenous DNA damage. *Nat Cancer* **2**, 643–657 (2021).

4. Nik-Zainal, S. *et al.* Landscape of somatic mutations in 560 breast cancer whole-genome sequences. *Nature* **534**, 47–54 (2016).

5. Degasperi, A. *et al.* A practical framework and online tool for mutational signature analyses show inter-tissue variation and driver dependencies. *Nat Cancer* **1**, 249–263 (2020). 6. Alexandrov, L. B. *et al.* The repertoire of mutational signatures in human cancer. *Nature* **578**, 94–101 (2020).

7. Liu, M., Katsevich, E., Janson, L. & Ramdas, A. Fast and powerful conditional randomization testing via distillation. *Biometrika* **109**, 277–293 (2022).
