# Supplementary material for: Comprehensive repertoire of the chromosomal alteration and mutational signatures across 16 cancer types
Source: Nat Genet. 2026 Feb 13;58(3):570–81. doi: 10.1038/s41588-025-02474-x (PMC12987726; doi:10.1038/s41588-025-02474-x)
Supplement: Supplementary file 2 — Reporting Summary [file 41588_2025_2474_MOESM2_ESM.pdf]

nature portfolio

Corresponding author(s): David C. Wedge, Andreas J. Gruber and Richard Houlston

Last updated by author(s): Oct 29, 2025

Reporting Summary

Nature Portfolio wishes to improve the reproducibility of the work that we publish. This form provides structure for consistency and transparency in reporting. For further information on Nature Portfolio policies, see our Editorial Policies and the Editorial Policy Checklist.

Statistics

For all statistical analyses, confirm that the following items are present in the figure legend, table legend, main text, or Methods section.

n/a	Confirmed	
☐	☒	The exact sample size (*n*) for each experimental group/condition, given as a discrete number and unit of measurement
☐	☒	A statement on whether measurements were taken from distinct samples or whether the same sample was measured repeatedly
☐	☒	The statistical test(s) used AND whether they are one- or two-sided<br>*Only common tests should be described solely by name; describe more complex techniques in the Methods section.*
☐	☒	A description of all covariates tested
☐	☒	A description of any assumptions or corrections, such as tests of normality and adjustment for multiple comparisons
☐	☒	A full description of the statistical parameters including central tendency (e.g. means) or other basic estimates (e.g. regression coefficient) AND variation (e.g. standard deviation) or associated estimates of uncertainty (e.g. confidence intervals)
☐	☒	For null hypothesis testing, the test statistic (e.g. *F*, *t*, *r*) with confidence intervals, effect sizes, degrees of freedom and *P* value noted<br>*Give P values as exact values whenever suitable.*
☒	☐	For Bayesian analysis, information on the choice of priors and Markov chain Monte Carlo settings
☐	☒	For hierarchical and complex designs, identification of the appropriate level for tests and full reporting of outcomes
☐	☒	Estimates of effect sizes (e.g. Cohen's *d*, Pearson's *r*), indicating how they were calculated

Our web collection on statistics for biologists contains articles on many of the points above.

Software and code

Policy information about availability of computer code

Data collection	Samples were collected and processed by Genomics England. The code used for curation of samples is available inside the Genomics England Research Environment under /re_gecip/shared_allGeCIPs/pancancer_signatures/code/processClinicalData. Software used for generating mutation matrices is detailed in supplementary table 13.
Data analysis	Software used for signature extraction is provided in supplementary table 13. Code used to perform subsequent data analysis is inside the Genomics England Research Environment. The code has been exported and published on GitHub at https://github.com/Wedge-lab/Gel_pan_cancer_signatures

For manuscripts utilizing custom algorithms or software that are central to the research but not yet described in published literature, software must be made available to editors and reviewers. We strongly encourage code deposition in a community repository (e.g. GitHub). See the Nature Portfolio guidelines for submitting code & software for further information.

Data

Policy information about availability of data

All manuscripts must include a data availability statement. This statement should provide the following information, where applicable:

- Accession codes, unique identifiers, or web links for publicly available datasets
- A description of any restrictions on data availability
- For clinical datasets or third party data, please ensure that the statement adheres to our policy

Data supporting study findings have been deposited in the National Genomic Research Library and can be accessed via the Genomics England Research Environment secure cloud workspace. The raw data, including patient profiles and corresponding genomic sequencing data, are available under restricted access for patient privacy reasons. Access can be obtained by first applying to become a member of either the Genomics England Research Network or the Discovery Forum (industry partners). The process for joining the network is described at https://www.genomicsengland.co.uk/join-us. The processed clinical and genomic data applied to the investigation are available in the Research Environment within the folder /re_gecip/shared_allGeCIPs/pancancer_signatures. At present, there is no proposed end date for data access within the research environment. All other public/private datasets used in the study, including corresponding download links and version numbers, can be found in Supplementary Tables.

Research involving human participants, their data, or biological material

Policy information about studies with human participants or human data. See also policy information about sex, gender (identity/presentation), and sexual orientation and race, ethnicity and racism.

Reporting on sex and gender	Sex was used as reported by NHSD, PHE/NCRAS and the GMCs where this matched the inferred sex from genomic sequencing. Where they do not match the sample was excluded.
Reporting on race, ethnicity, or other socially relevant groupings	Reported race, ethnicity, or other socially relevant groupings were not used in this study. Principal components of germline genetic variants were used to control for population structure as described in the manuscript and methods.
Population characteristics	Information on the age distribution of tumour groups is provided in supplementary table 1. The collection and processing of treatment information is described in detail in the methods. The exposure of patients to clinical treatments such as chemotherapy and radiotherapy is used in this study. However this information cannot be exported from the research environment to protect the identity of participants. This information is available to researchers within the Genomics England Research Environment under /re_gecip/shared_allGeCIPs/pancancer_signatures/results/.
Recruitment	Clinical and demographic data were obtained from NHS Digital (NHSD), Public Health England's National Cancer Registration and Analysis Service (PHE-NCRAS) and the Genomic Medicine Centres (GMCs) through the Genomics England Research Environment.
Ethics oversight	The 100,000 Genomes Project protocol was approved by the East of England and South Cambridge Research Ethics Committee on 20 February 2015, REC reference 14/EE/1112

Note that full information on the approval of the study protocol must also be provided in the manuscript.

Field-specific reporting

Please select the one below that is the best fit for your research. If you are not sure, read the appropriate sections before making your selection.

☒ Life sciences ☐ Behavioural & social sciences ☐ Ecological, evolutionary & environmental sciences

For a reference copy of the document with all sections, see nature.com/documents/nr-reporting-summary-flat.pdf

Life sciences study design

All studies must disclose on these points even when the disclosure is negative.

Sample size	10983 samples were included in the full cohort. Exact sample sizes for tumour groups are provided in supplementary table 2.
Data exclusions	A detailed description of the sample quality control is provided in the methods. Supplementary table 1 provides information on how many samples were excluded. Sequenced tumour samples were excluded if clinical data were missing or if unresolvable conflicts existed between the clinical data sources (GMCs, NHSD, PHE-NCRAS, histology reports). In total 2,251/14,129 (15.9%) of tumour samples were excluded based on availability and consistency of reported sex, tumour histology, tumour type, sampling date or if the participant was recorded as less than 18 years old at the time of sampling. 267/11878 (2.2%) of tumour samples with required clinical data available were excluded based on tumour sample purity and sequencing data quality. Duplicate tumour samples were also removed, to ensure that no individual was represented more than once in a tumour group. If multiple sequenced tumour samples from the same tumour group were available for an individual, we preferentially kept primary tumour samples with highest purity. Based on these criteria, 10,983 tumour samples were suitable for analysis.
Replication	This study has an observational rather than an experimental study design, and only one sample was sequenced from each participant, in the

Replication	great majority of cases.<br>Mutational signatures were extracted using SigProfilerExtractor. We replicate many of the findings previously published for PCAWG (Alexandrov et al. 2020) and TCGA (Steele et al. 2022) using the same code. We also reproduce results found using other NMF-based algorithms in many independent studies referenced in the manuscript. Results were also replicated between 16 tumour cohorts on which signature extraction was performed independently.
Randomization	Age, sex and population principal components were included as covariates in analysis to control for possible population stratification. Conditional randomisation testing was performed to generate null distributions by randomly resampling test variables.
Blinding	This study used real-world observation data collected from NHS trusts. The investigators did not have control over sample selection, collection and processing and as such blinding is not relevant to this study.

Reporting for specific materials, systems and methods

We require information from authors about some types of materials, experimental systems and methods used in many studies. Here, indicate whether each material, system or method listed is relevant to your study. If you are not sure if a list item applies to your research, read the appropriate section before selecting a response.

Materials & experimental systems

n/a	Involved in the study
☒	☐ Antibodies
☒	☐ Eukaryotic cell lines
☒	☐ Palaeontology and archaeology
☒	☐ Animals and other organisms
☒	☐ Clinical data
☒	☐ Dual use research of concern
☒	☐ Plants

Methods

n/a	Involved in the study
☒	☐ ChIP-seq
☒	☐ Flow cytometry
☒	☐ MRI-based neuroimaging
